# NALIRIFOX, FOLFIRINOX, and Gemcitabine With Nab-Paclitaxel as First-Line Chemotherapy for Metastatic Pancreatic Cancer: A Systematic Review and Meta-Analysis

**DOI:** 10.1001/jamanetworkopen.2023.50756

**Published:** 2024-01-08

**Authors:** Federico Nichetti, Simone Rota, Paolo Ambrosini, Chiara Pircher, Eleonora Gusmaroli, Michele Droz Dit Busset, Sara Pusceddu, Carlo Sposito, Jorgelina Coppa, Federica Morano, Filippo Pietrantonio, Maria Di Bartolomeo, Luigi Mariani, Vincenzo Mazzaferro, Filippo de Braud, Monica Niger

**Affiliations:** 1 Department of Medical Oncology, Fondazione IRCCS Istituto Nazionale dei Tumori, Milan, Italy; 2 Computational Oncology Group, Molecular Precision Oncology Program, National Center for Tumor Diseases and German Cancer Research Center , Heidelberg, Germany; 3 Hepato-Pancreato-Biliary Surgery and Liver Transplantation, Fondazione IRCCS Istituto Nazionale dei Tumori, Milan, Italy; 4 Department of Oncology and Hemato-Oncology, University of Milan, Milan, Italy; 5 Department of Epidemiology and Data Science, Fondazione IRCCS Istituto Nazionale dei Tumori di Milano, Milan, Italy

## Abstract

**Importance:**

The NAPOLI 3 trial showed the superiority of fluorouracil, leucovorin, liposomal irinotecan, and oxaliplatin (NALIRIFOX) over the combination of gemcitabine and nab-paclitaxel (GEM-NABP) as first-line treatment of metastatic pancreatic ductal adenocarcinoma (PDAC). Analyses comparing NALIRIFOX and GEM-NABP with fluorouracil, leucovorin, irinotecan, and oxaliplatin (FOLFIRINOX) have not yet been reported.

**Objective:**

To derive survival, response, and toxic effects data from phase 3 clinical trials and compare NALIRIFOX, FOLFIRINOX, and GEM-NABP.

**Data Sources:**

After a systematic search of PubMed, Scopus, Embase, and American Society of Clinical Oncology and European Society for Medical Oncology meetings’ libraries, Kaplan-Meier curves were extracted from phase 3 clinical trials conducted from January 1, 2011, until September 12, 2023.

**Study Selection:**

Phase 3 clinical trials that tested NALIRIFOX, FOLFIRINOX, or GEM-NABP as first-line treatment of metastatic PDAC and reported overall survival (OS) and progression-free survival (PFS) curves were selected. This study followed the Preferred Reporting Items for Systematic Reviews and Meta-analyses of Individual Participant Data reporting guidelines.

**Data Extraction And Synthesis:**

Individual patient OS and PFS data were extracted from Kaplan-Meier plots of original trials via a graphic reconstructive algorithm. Overall response rates (ORRs) and grade 3 or higher toxic effects rates were also collected. A pooled analysis was conducted, and results were validated via a network meta-analysis.

**Main Outcomes and Measures:**

The primary end point was OS. Secondary outcomes included PFS, ORR, and toxic effects rates.

**Results:**

A total of 7 trials with data on 2581 patients were analyzed, including 383 patients treated with NALIRIFOX, 433 patients treated with FOLFIRINOX, and 1756 patients treated with GEM-NABP. Median PFS was longer in patients treated with NALIRIFOX (7.4 [95% CI, 6.1-7.7] months) or FOLFIRINOX (7.3 [95% CI, 6.5-7.9] months; [HR], 1.21 [95% CI, 0.86-1.70]; *P* = .28) compared with patients treated with GEM-NABP (5.7 [95% CI, 5.6-6.1] months; HR vs NALIRIFOX, 1.45 [95% CI, 1.22-1.73]; *P* < .001). Similarly, GEM-NABP was associated with poorer OS (10.4 [95% CI, 9.8-10.8]; months) compared with NALIRIFOX (HR, 1.18 [95% CI, 1.00-1.39]; *P* = .05], while no difference was observed between FOLFIRINOX (11.7 [95% CI, 10.4-13.0] months) and NALIRIFOX (11.1 [95% CI, 10.1-12.3] months; HR, 1.06 [95% CI, 0.81-1.39]; *P* = .65). There were no statistically significant differences in ORR among NALIRIFOX (41.8%), FOLFIRINOX (31.6%), and GEM-NABP (35.0%). NALIRIFOX was associated with lower incidence of grade 3 or higher hematological toxic effects (eg, platelet count decreased 1.6% vs 11.8% with FOLFIRINOX and 10.8% with GEM-NABP), but higher rates of severe diarrhea compared with GEM-NABP (20.3% vs 15.7%).

**Conclusions and Relevance:**

In this systematic review and meta-analysis, NALIRIFOX and FOLFIRINOX were associated with similar PFS and OS as first-line treatment of advanced PDAC, although NALIRIFOX was associated with a different toxicity profile. Careful patient selection, financial toxic effects consideration, and direct comparison between FOLFIRINOX and NALIRIFOX are warranted.

## Key Points

**Question:**

Does fluorouracil, leucovorin, liposomal irinotecan and oxaliplatin (NALIFIROX) confer a survival benefit as first-line treatment for patients with metastatic pancreatic cancer compared with fluorouracil, leucovorin, irinotecan, and oxaliplatin (FOLFIRINOX) or gemcitabine with nab-paclitaxel (GEM-NABP)?

**Findings:**

In this systematic review and analysis of 7 phase 3 clinical trials with 2581 patients testing first-line NALIRIFOX, FOLFIRINOX or GEM-NABP for metastatic pancreatic cancer, NALIFIROX and FOLFIRINOX showed superior efficacy in terms progression-free and overall survival compared with GEM-NABP, although no difference was highlighted between NALIFIROX and FOLFIRINOX. NALIRIFOX was associated with lower incidence of hematological events, but significantly higher rates of severe diarrhea compared with both other regimens.

**Meaning:**

These findings suggest that NALIRIFOX and FOLFIRINOX may provide equal efficacy as first-line treatment of metastatic pancreatic cancer, but with different toxicity profiles.

## Teaser

This systematic review and meta-analysis compares survival, response, and toxic effects among patients with pancreatic cancer treated with fluorouracil, leucovorin, liposomal irinotecan and oxaliplatin (NALIFIROX); fluorouracil, leucovorin, irinotecan, and oxaliplatin (FOLFIRINOX); or gemcitabine with nab-paclitaxel.

## Introduction

Combination chemotherapy represents the standard of care for advanced pancreatic ductal adenocarcinoma (PDAC). In particular, FOLFIRINOX, consisting of fluorouracil, leucovorin, irinotecan, and oxaliplatin,^1^ or gemcitabine with nab-paclitaxel (GEM-NABP)^2^ have long represented the gold standard first-line treatment in patients with metastatic disease, as both regimens were proven superior to gemcitabine monotherapy. To our knowledge, these combinations have never been formally compared in a clinical trial, so observational studies and indirect evaluations (eg, meta-analyses) have tried to define which patients could benefit most from each regimen.^3,4^ Recently, several investigational agents alone or in combination with standard chemotherapy (mostly with GEM-NABP) have been tested, all failing to demonstrate a benefit in phase 3 clinical trials.

In this context, the 2023 NAPOLI 3 trial^5^ was the first positive phase 3 trial in this setting in a decade. The study compared the combination of fluorouracil, leucovorin, liposomal irinotecan, and oxaliplatin (NALIRIFOX) with GEM-NABP, showing a benefit of the NALIRIFOX regimen in terms of both PFS and OS and thus becoming a candidate as a new reference regimen in this setting.^5^ However, while NALIRIFOX and FOLFIRINOX share a similar chemotherapy profile, they are unlikely to be directly compared for efficacy and tolerability in a clinical trial.

Based on these considerations, we performed a systematic review and meta-analysis of phase 3 clinical trials of first-line treatment of metastatic PDAC, with the aim of comparing GEM-NABP, FOLFIRINOX, and NALIRIFOX in terms of PFS, OS, response rates, and toxicity profiles.

## Methods

### Study Selection Procedure

We performed a reconstructed individual patient data (IPD) pooled analysis of phase 3 clinical trials and validated our results by means of a network meta-analysis (NMA) of selected studies. The Preferred Reporting Items for Systematic Reviews and Meta-analyses (PRISMA) reporting guidelines for IPD (PRISMA-IPD) and for NMA (PRISMA-NMA) were followed.

To this aim, we selected studies adopting the following criteria: phase 3 clinical trials; patients with metastatic PDAC (excluding locally advanced, unresectable PDAC); first-line treatment; at least 1 trial group (experimental and/or control) receiving GEM-NABP, FOLFIRINOX, or NALIRIFOX, planned at standard dose density and intensity; and available PFS and OS Kaplan-Meier plots with number-at-risk tables. Studies testing GEM-NABP, FOLFIRINOX or NALIRIFOX at 50% or lower doses were excluded. Prior adjuvant treatment was allowed, according to each trial inclusion criteria.

A systematic review was conducted on PubMed, Scopus, Embase, and American Society of Clinical Oncology and European Society for Medical Oncology meetings’ libraries for eligible studies performed between January 1, 2011, and September 12, 2023. In the screening procedure, 2 reviewers (F.N. and S.R.) independently searched and selected abstracts according to the search criteria. The query string for each database is provided in the eMethods in Supplement 1. If either of the studies was reported more than once with updated results, only the latest and most complete publication was used as the primary trial source. The trials were assessed for risk of bias by using the Cochrane Risk of Bias (version 2) tool for randomized clinical trials (RCTs).^6^

For each eligible study, background information was extracted for the trial’s design, inclusion and exclusion criteria, treatment regimens (dose and schedule), number of patients, and baseline clinical features. Moreover, the absolute numbers according to best response and patients experiencing grade 3 or higher toxic effects were collected.

### Reconstruction of Time-to-Event Outcomes

A graphical reconstructive algorithm was used to estimate time-to-event outcomes (OS and PFS) from reported Kaplan-Meier plots of each group of each study according to the method by Guyot et al^7^ and implemented by Liu et al,^8^ as previously reported.^9^ Data reconstruction was performed independently by 3 investigators (F.N., S.R., and P.A.) and the best reconstruction was selected. Details about reconstruction accuracy evaluation are reported in the eMethods in Supplement 1.

Once extracted, IPD of the same treatment group (GEM-NABP, FOLFIRINOX, or NALIRIFOX) across different trials were pooled. Other comparator groups were removed and used only in the validation NMA.

### Statistical Analysis

The primary end point of the analysis was OS, as evaluated as the time from treatment start to death or last follow-up within the range of observation periods in the clinical trials included, for each treatment group. Secondary end points were PFS, evaluated as the time from treatment start to disease progression, death, or last follow-up within the range of observation periods in the clinical trials included; overall response rate (ORR), defined as the rate of patients experiencing complete or partial response out of all patients in each treatment group; and the rate of grade 3 or higher toxic effects for each treatment group. Studies lacking detailed information about the number of patients evaluable for treatment response and studies not reporting the detailed number of patients experiencing a specific toxic effect were excluded from their respective analyses.

To validate the pooled analysis survival results despite a possible bias due to different median follow-up times among included trials, 3 approaches were adopted: (1) 16- and 12-month PFS and OS rates were evaluated; (2) in a secondary analysis, reconstructed survival data were censored at the time of the shortest follow-up among included studies; and (3) a frequentist method-based NMA was performed using hazard ratios (HRs) from the original trials.

Furthermore, to determine the power and potential sample size required to appropriately demonstrate significant of PFS and OS findings, power analyses were performed using estimated treatment effects from the Cox proportional hazards models of derived subgroups. To estimate the power of our analysis, together with the required 1:1 sample size to demonstrate NALIRIFOX as the superior regimen with 80% power, we further pooled the treatment groups into experimental (NALIRIFOX) and control (FOLFIRINOX and GEM-NABP) and evaluated the study power using the HR of the comparison together with α = 5%. Similarly, sensitivity analyses were performed by excluding the FOLFIRINOX and GEM-NABP groups in turn and comparing with NALIRIFOX. In case of similar treatment outcomes between 2 regimens (ie, HRs between 0.90 and 1.10), a noninferiority design was adopted.

Median follow-up was quantified with the reverse Kaplan-Meier estimator, while pooled PFS and OS curves were estimated with the Kaplan-Meier method, and all were compared by means of global and pairwise log-rank tests. The outcome of each group was investigated with Cox proportional hazard regression models, with individual patient’s clinical trial data included as a random variable to account for interstudy differences, as in previous works.^10,11^ The methods are further detailed in the eMethods in Supplement 1.

Model results were summarized using HRs, together with the corresponding 95% CIs and likelihood ratio test *P* values. We compared 6- and 12-month survival rates using Peto and Peto modification of Gehan Wilcoxon test to account for early differences in survival times. The NMAs was conducted based on a 1-stage, frequentist approach to calculate the pooled effect estimates for all interventions compared with the reference treatment (NALIRIFOX), by means of random-effects models. Given the design of included trials, neither within-designs heterogeneity (ie, only 1 comparison per design) nor between-designs inconsistency (ie, no loops in the network) could be identified.

Pooled rates of grade 3 or higher toxic effects and best response rates among different treatment groups were compared using χ^2^ tests. Moreover, logistic regression was used to assess the probability of grade 3 or higher toxic effects and of response to treatment, with individual patient’s clinical trial included as a random variable. Of note, equivalent toxic effects terms reported separately in original reports were pooled, namely *neutrophil count decreased* and *neutropenia*, *peripheral neuropathy* and *peripheral sensory neuropathy*, and *fatigue* and *asthenia*.

The threshold for statistical significance was set to *P* = .05 and all statistical tests were 2-sided. All analyses were conducted using R statistical software version 4.2.2 (R Project for Statistical Computing). A full list of R packages used in analyses is provided in the eMethods in Supplement 1. Data were analyzed from June 1 to September 12, 2023.

## Results

### Study Selection and Characteristics

**Figure 1.**
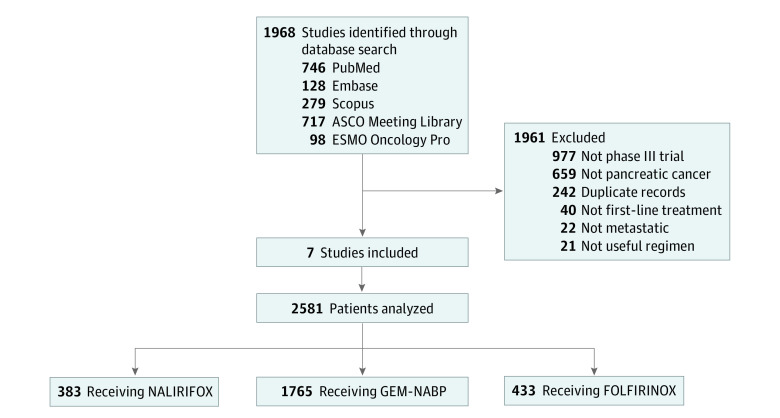
Study Selection Flowchart ASCO indicates American Society of Clinical Oncology; ESMO, European Society for Medical Oncology; FOLFIRINOX, irinotecan, oxaliplatin, folinic acid, and fluoruracil; GEM-NABP, gemcitabine and nab-paclitaxel; NALIRIFOX, liposomal irinotecan, oxaliplatin, folinic acid, and fluoruracil.

A total of 1968 studies were screened by title and abstract, and 7 studies^1,2,5,12,13,14,15^ with IPD for 2581 participants were included in the main analysis (Figure 1; eFigure 1 in Supplement 1). By treatment group, 383 participants (14.8%) were treated with NALIRIFOX, 1765 participants (68.4%) were treated with GEM-NABP, and 433 participants (16.8%) were treated with FOLFIRINOX.

The study selection process is shown in Figure 1 and eFigure 1 in Supplement 1. Of note, 1 trial^16^ was excluded despite including FOLFIRINOX in the required setting because treatment was administered at significantly lower doses than standard of care. Of 7 trials^1,2,5,12,13,14,15^ included in analysis, 2 studies (ACCORD 11^1^ and AVENGER500^12^) included FOLFIRINOX (as experimental and control groups, respectively), while the remaining 5 trials all had GEM-NABP, which represented the experimental group only in the MPACT trial.^2^ As expected, NALIRIFOX was tested only in the NAPOLI 3 trial.^5^ The characteristics of the studies are summarized in the Table and eTable 1 in Supplement 1.

**Table.** Summary of Clinical Trials Characteristics

Trial name	NAPOLI 3^5^	ACCORD 11^1^	MPACT^2^	HALO^14^	RESOLVE^15^	AVENGER500^12^	CanStem111P^13^
ClinicalTrials.gov identifier	NCT04083235	NCT00112658	NCT00844649	NCT02715804	NCT02436668	NCT03504423	NCT02993731
Study timeframe	February 2020 to July 2022	December 2005 to October 2009	May 2009 to April 2012	March 2016 to December 2018	May 2015 - October 2018	November 2018 to January 2022	January 2017 to February 2019
Publication year	2023	2011	2013	2020	2021	Presented at ASCO 2022	2023
Geographic area	Global	France	Global	Global	Global	Global	Global
Blinding	Open-label	Open-label	Open-label	Double-blind	Double-blind	Open-label	Open-label
Randomization	1:1	1:1	1:1	2:1	1:1	1:1	1:1
Primary end point	OS	OS	OS	OS	OS, PFS	OS	OS
Secondary end points	PFS, ORR	PFS, ORR, safety, QOL	PFS and ORR	PFS, ORR, safety	ORR, CA 19-9 response, QOL, safety	PFS, DOR, QOL, safety	PFS, DCR, ORR
Total patients enrolled, No.	770	342	861	492	424	528	1134
Experimental group	NALIRIFOX (n = 383)	FOLFIRINOX (n = 171)	GEM-NABP (n = 431)	PEGPH20 + GEM- NABP (n = 327)	Ibrutinib + GEM-NABP (n = 211)	Demivistat + FOLFIRINOX (n = 266)	Napabucasin + GEM-NABP (n = 565)
Details	Liposomal irinotecan 50 mg/m^2^ + oxaliplatin 60 mg/m^2^ + LV 400 mg/m^2^ +fluorouracil 2400 mg/m^2^ over 46 h; every 15 d	Irinotecan 180 mg/m^2^ + oxaliplatin 85 mg/m^2^ + LV 400 mg/m^2^ +fluorouracil bolus 400 mg/m^2^ +2400 mg/m^2^ over 46 h; every 15 d	GEM 1000 mg/m^2^ + NABP 125 mg/m^2^; Days 1, 8, 15, and every 28	3.0 μg/kg of PEGPH20 as IV infusion, 2/wk for wk 1-3 of cycle 1, then 1/wk for wk 1-3 of cycle 2 and beyond in combination with GEM-NABP	Ibrutinib 560 mg once daily + Gem 1000 mg/m^2^ + NABP 125 mg/m^2^; days 1, 8, 15, and 28	Devimistat 500 mg/m^2^ on days 1 and 3 + irinotecan 140 mg/m^2^ + oxaliplatin 65 mg/m^2^ + LV 400 mg/m^2^ +fU bolus 400 mg/m^2^ +2400 mg/m^2^ over 46 h; every 15 d	Napabucasin 240 mg 2/d + GEM 1000 mg/m^2^ + NABP 125 mg/m^2^; days 1, 8, 15, and every 28
Control group	GEM-NABP (n = 387)	GEM (n = 171)	GEM (n = 430)	GEM-NABP (n = 165)	GEM-NABP (n = 213)	FOLFIRINOX (n = 262)	GEM-NABP (n = 569)
Details	GEM 1000 mg/m^2^ + NABP 125 mg/m^2^; days 1, 8, 15, and every 28	GEM 1000 mg/m^2^; cycle 1: weekly (7 of 8 wk); cycle 2 onward: days 1, 8, 15, and every 28	GEM 1000 mg/m^2^; cycle 1: weekly (7 of 8 wk); cycle 2 onward: days 1, 8, 15, and every 28	GEM 1000 mg/m^2^ + NABP125 mg/m^2^; days 1, 8, 15, and every 28	GEM 1000 mg/m^2^ + NABP 125 mg/m^2^; days 1, 8, 15, and every 28	Irinotecan 180 mg/m^2^ + oxaliplatin 85 mg/m^2^ + LV 400 mg/m^2^ +fluorouracil bolus 400 mg/m^2^ +2400 mg/m^2^ over 46 h; every 15 d	GEM 1000 mg/m^2^ + NABP 125 mg/m^2^; days 1, 8, 15, and every 28
Treatment duration	Until disease progression or unacceptable toxic effects	6 mo recommended for patients who had a response, continuation and/or reintroduction allowed	Until disease progression or unacceptable toxic effects	Until disease progression or unacceptable toxic effects	Until disease progression or unacceptable toxic effects	Until disease progression or unacceptable toxic effects	Until disease progression or unacceptable toxic effects
Time since metastatic disease diagnosis	≤6 wk	Not specified	≤6 wk	Not specified	≤6 wk	Not specified	Not specified
Type of metastatic lesions	RECIST criteria, version 1.1	RECIST criteria, version 1.0	RECIST criteria, version 1.0	RECIST criteria, version 1.1	RECIST criteria, version 1.1	RECIST criteria, version 1.1	RECIST criteria, version 1.1
Other relevant criteria	NA	NA	NA	Hyaluronan-high tumors, defined as ≥50% hyaluronan staining in the ECM of tumor samples	NA	NA	NA
Neoadjuvant or adjuvant chemotherapy	Allowed (>12 mo before trial treatment)	Not allowed	Fluorouracil or GEM as radiation sensitizer in the adjuvant setting (>6 mo before trial treatment)	Allowed (>6 mo before trial treatment)	Not allowed	Allowed	Not allowed
Age limit	None	76 y	None	None	None	75 y	None

Abbreviations: ASCO, American Society of Clinical Oncology; CA 19-9, carbohydrate antigen 19.9; DCR, disease control rate; DOR, duration of response; ECM, extracellular matrix; FOLFIRINOX, fluorouracil, leucovorin, irinotecan, and oxaliplatin; GEM, gemcitabine; LV, leucovorin; NA, not applicable; NABP, nab-paclitaxel; NALIRIFOX, fluorouracil, leucovorin, liposomal irinotecan and oxaliplatin; ORR, overall response rate; OS, overall survival; PEGPH20, pegvorhyaluronidase alfa; PFS, progression free survival; QOL, quality of life; RECIST, Response Evaluation Criteria in Solid Tumours.

The risk of bias analysis yielded low risk for all studies (eTable 2 in Supplement 1). The graphical reconstructive algorithm yielded patient-level data that derived similar median PFS, OS, and HRs to original trials. Furthermore, a near-complete overlap was observed in survival curves compared with matched cohorts in the original plots (eTable 3 in Supplement 1).

### Survival Outcomes and ORRs

Median (IQR) follow-up was 18.8 (13.6-23.5) months overall, and 16.2 (13.5-18.9) months in the NALIRIFOX group, 20.3 (13.7-24.6) months for the pooled GEM-NABP group, and 18.8 (13.3-23.8) months for the pooled FOLFIRINOX group. Pairwise comparison between treatment groups revealed that median follow up times were significantly shorter for the NALIRIFOX group (log-rank *P* vs GEM-NABP < .001 and log-rank *P* vs FOLFIRINOX < .009), while no significant difference was found between FOLFIRINOX and GEM-NABP (log-rank *P* = .30).

**Figure 2.**
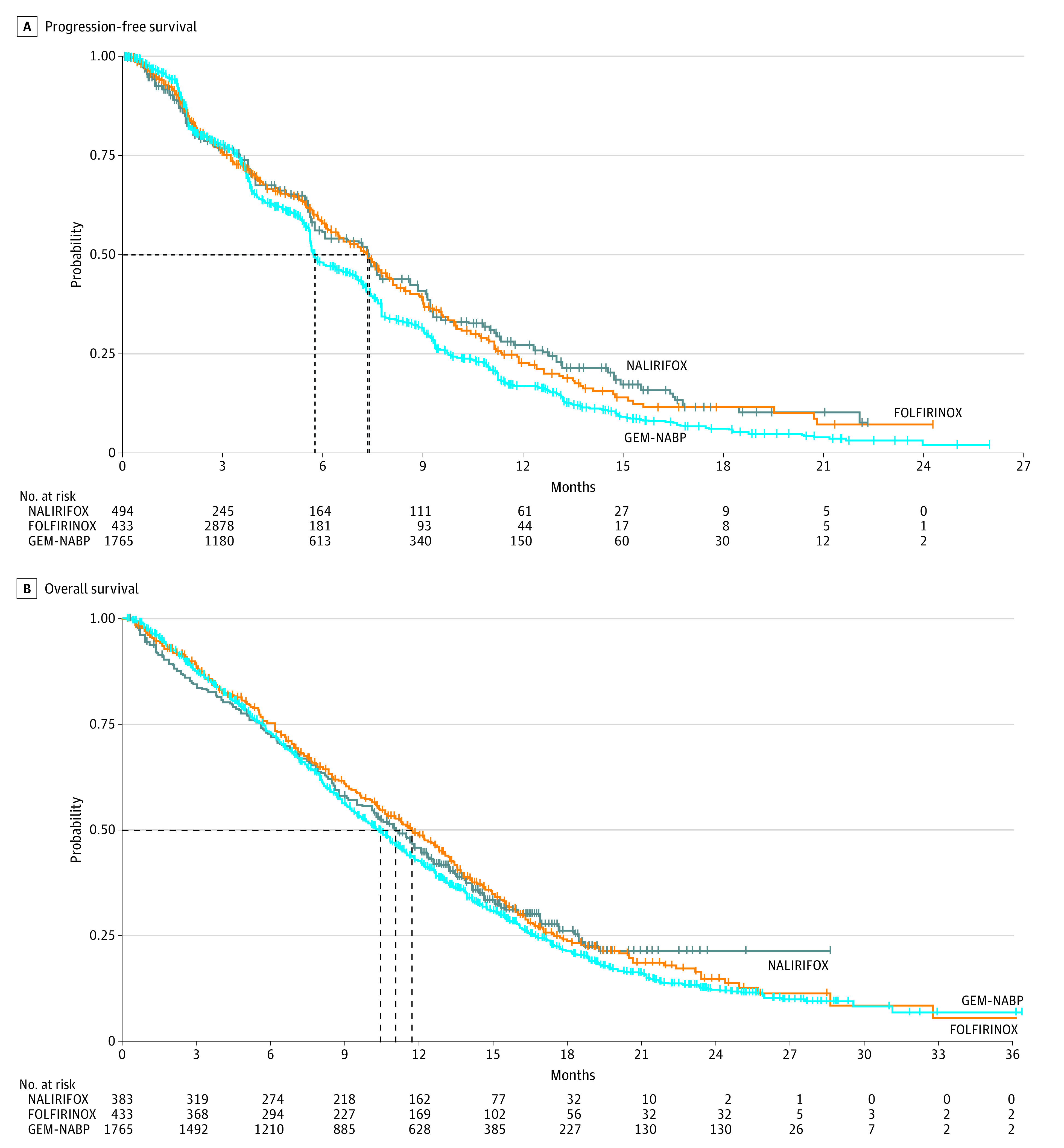
Reconstructed Kaplan-Meier Plots for Progression-Free Survival and Overall Survival According to First-Line Regimen FOLFIRINOX indicates irinotecan, oxaliplatin, folinic acid, and fluoruracil; GEM-NABP, gemcitabine and nab-paclitaxel; NALIRIFOX, liposomal irinotecan, oxaliplatin, folinic acid, and fluoruracil. Dotted lines indicate median survival.

Median PFS was 7.4 (95% CI, 6.1-7.7) months for NALIRIFOX, 5.7 (95% CI, 5.6-6.1) months for GEM-NABP and 7.3 (95% CI, 6.5-7.9) months for FOLFIRINOX (global log-rank *P* < .001) (Figure 2A; eTable 4 in Supplement 1). Using NALIRIFOX as the reference group and accounting for between-study heterogeneity, the GEM-NABP group had worse PFS (HR, 1.45 [95% CI, 1.22-1.73]; *P* < .001), while no statistically significant difference was observed for the FOLFIRINOX group (HR, 1.21 [95% CI, 0.86-1.70]; *P* = .28).

Median OS was 11.1 (95% CI, 10.1-12.3) months for NALIRIFOX, 10.4 (95% CI, 9.8-10.8) months for GEM-NABP, and 11.7 (95% CI, 10.4-13.0) months for FOLFIRINOX (global log-rank *P* = .19) (Figure 2B; eTable 4 in Supplement 1). Compared with NALIRIFOX, GEM-NABP was associated with worse OS (HR, 1.18 [95% CI, 1.00-1.39]; *P* = .05) but there was no significant difference for FOLFIRINOX (HR, 1.06 [95% CI, 0.81-1.39]; *P* = .65).

These results were confirmed in a secondary analysis censored at the shortest median follow up (ie, 16.2 months) (eFigure 2 in Supplement 1). Moreover, using the NMA approach, GEM-NABP was confirmed as having significantly inferior PFS (HR, 1.45 [95% CI, 1.21-1.73]; *P* < .001) and OS (HR, 1.20 [95% CI, 1.01-1.43]; *P* = .03) compared with NALIRIFOX, while no significant difference was observed with FOLFIRINOX (PFS: HR, 0.99 [95% CI, 0.70-1.39]; *P* = .94; OS: HR, 0.95 [95% CI, 0.68-1.33]; *P* = .78) (eFigure 3 in Supplement 1).

Analysis of 6- and 12-month OS did not find statistically significantly higher OS for NALIRIFOX compared with FOLFIRINOX or GEM-NABP. Analysis of 6- and 12-month PFS found significantly lower PFS for GEM-NABP compared with both NALIRIFOX and FOLFIRINOX (eTable 5 in Supplement 1).

Furthermore, as an exploratory analysis, the differences in PFS and OS of patients treated with GEM-NABP across different trials were tested. Notably, the outcomes associated with GEM-NABP improved over the years with each trial, while the GEM-NABP group of the NAPOLI 3 trial^5^ reported no significant differences in OS or PFS compared with the MPACT study^2^ (eFigure 4 in Supplement 1).

In terms of response rates, the AVENGER500^12^ and RESOLVE^15^ trials did not report detailed absolute numbers and percentages regarding treatment response and were thus excluded from our ORR analysis. According to the remaining trials, there was no statistically significant difference in ORR for NALIRIFOX (41.8%) compared with FOLFIRINOX (31.6%) or GEM-NABP (35.0%) (NALIRIFOX vs FOLFIRINOX: adjusted odds ratio [aOR], 1.45 [95% CI, 0.67-3.11], *P* = .34; NALIRIFOX vs GEM-NABP: aOR, 1.28 [95% CI, 0.96-1.70]; *P* = .96; FOLFIRINOX vs GEM-NABP: aOR, 0.88 [95% CI, 0.43-1.83]; *P* = .74). Detailed ORR findings are reported in eTable 1 and eFigure 5 in Supplement 1.

### Power Analysis

Given the very similar outcomes observed between cohorts treated with NALIRIFOX and FOLFIRINOX, a superiority analysis design would require an unrealistic number of patients to demonstrate a very small difference. Therefore, power and sample size evaluations of this comparison were performed using a noninferiority design. The evaluable noninferiority margin (with 80% power and with the observed sample size and with the probability of event observed in upstream analyses approximately 70%) and the sample size required to assess noninferiority (with the boundary set as the reciprocal of the HR observed between NALIRIFOX and GEM-NABP) were tested.

Results are provided in eTable 6 in Supplement 1. The analysis confirmed that our study had sufficient power to compare NALIRIFOX with GEM-NABP in terms of PFS, although less power in terms of OS (approximately 65%), given the unbalanced sizes of the groups and the smaller effect size. However, based on our evidence, a clinical trial testing this end point would require a large number of patients (approximately 800 patients per group). Concerning NALIRIFOX vs FOLFIRINOX, the sample size in this systematic review and meta-analysis (ie, 383 patients in the NALIFRIFOX groups vs 433 patients in the FOLFIRINOX group) allows us to demonstrate, with 80% power, a noninferiority margin up to 1.23 (ie, FOLFIRINOX would be considered noninferior if the HR vs NALIRIFOX did not exceed 1.23) both for OS and for PFS. In contrast, taking the reciprocal of the observed HR for the NALIRIFOX vs GEM-NABP comparison as the margin (OS, 1.18; PFS, 1.43), a noninferiority study would similarly require a very large number of patients (approximately 1400 patients) for OS, while the sample size needed for PFS (287 patients) would be smaller than our actual cohort.

### Safety

**Figure 3.**
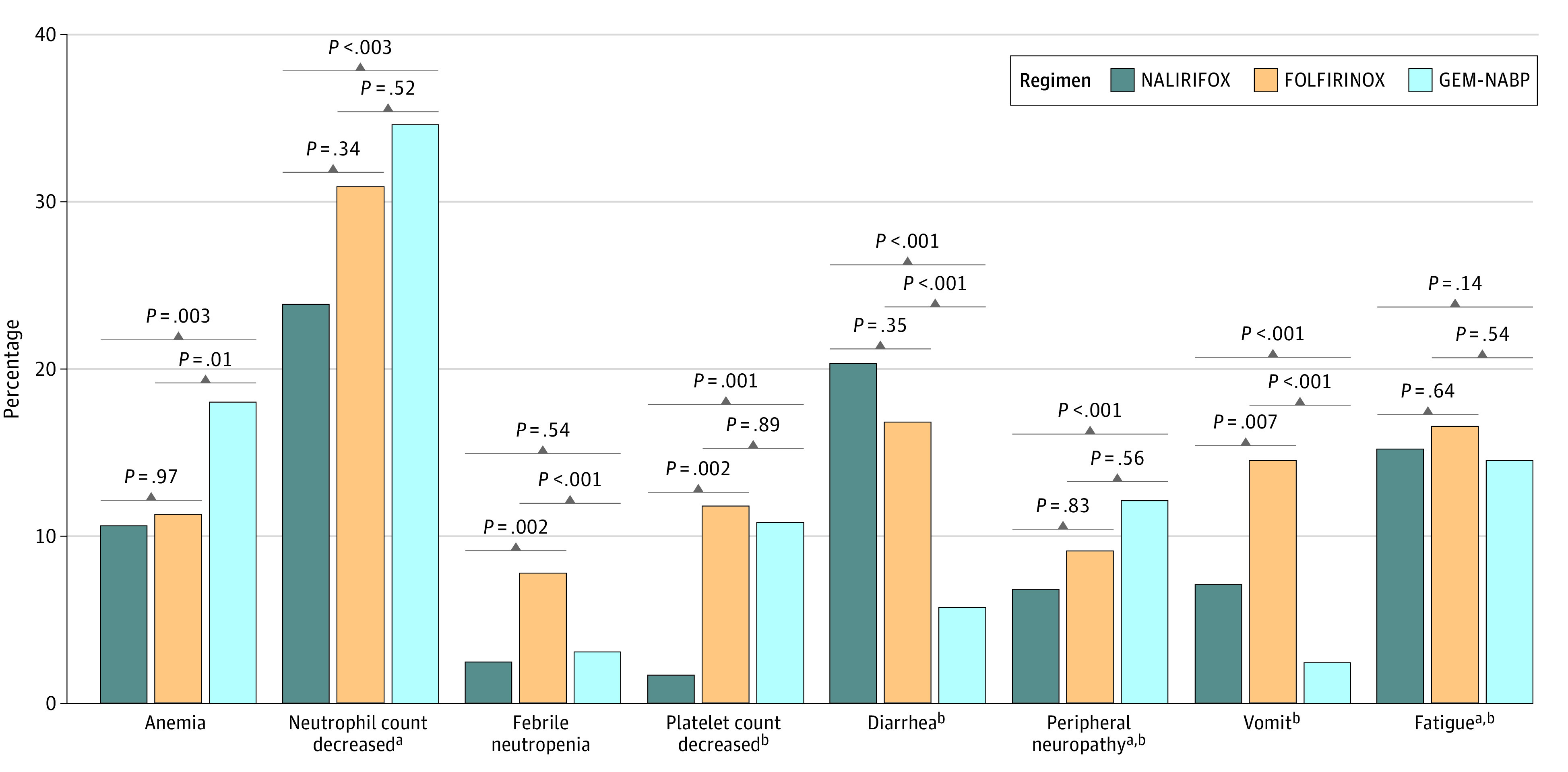
Reporting Incidence of Grade 3 or Higher Toxic Effects According to the Pooled Treatment Regimens *P* values of adjusted logistic regression models are plotted for each comparison. FOLFIRINOX indicates irinotecan, oxaliplatin, folinic acid, and fluoruracil; GEM-NABP, gemcitabine and nab-paclitaxel; NALIRIFOX, liposomal irinotecan, oxaliplatin, folinic acid, and fluoruracil. ^a^Equivalent terms reported separately in original reports were pooled before the analysis, including *neutrophil count decreased* and *neutropenia*, *peripheral neuropathy* and *peripheral sensory neuropathy*, and *fatigue* and *asthenia*. ^b^The following toxic effects were not detailed in all trials: platelet count decreased and fatigue rates were not available in CanStem111P^13^ trial results; diarrhea rates were not available in HALO trial^14^ results; peripheral neuropathy rates were not available in CanStem111P,^13^ HALO,^14^ and AVENGER500^12^ trial results; vomit rates were not available in CanStem111P, MPACT,^2^ HALO,^14^ and AVENGER500^12^ trial results.

We compared the 3 pooled regimens in terms of toxic effects. Details on missingness of toxic effects data in each trial are provided in Figure 3 and eTable 7 in Supplement 1. Overall, NALIRIFOX was associated with significantly lower incidence of thrombocytopenia compared with both other regimens (1.6% vs 11.8% with FOLFIRINOX and 10.8% with GEM-NABP), and of anemia and neutropenia compared with GEM-NABP. A higher incidence of diarrhea was reported with NALIRIFOX (20.3%) vs GEM-NABP (15.7%), although not significantly more than in patients treated with FOLFIRINOX (16.8%). Conversely, FOLFIRINOX was associated with the highest risk of febrile neutropenia and vomiting, while patients receiving GEM-NABP reported the highest rates of anemia and peripheral neuropathy compared with the other regimens.

## Discussion

This systematic review and meta-analysis compared NALIRIFOX, FOLFIRINOX, and GEM-NABP in terms of survival outcomes, response rates, and toxic effects from phase 3 trials. Our findings suggest that NALIRIFOX and FOLFIRINOX may provide equal efficacy as first-line treatment of metastatic PDAC but with different toxicity profiles. The treatment of metastatic PDAC remains a significant challenge in oncology, as the most used regimens, FOLFIRINOX and GEM-NABP, have moderate efficacy in terms of PFS and OS, which are still often less than 1 year. Overall, FOLFIRINOX has historically been reported to provide higher ORR and superior survival outcomes at the cost of greater toxic effects compared with GEM-NABP, although without a formal head-to-head comparison.^3^ As a result, there is heterogeneity in the choice of the appropriate first-line of treatment in daily clinical practice, with GEM-NABP administered to a wider patient population, while FOLFIRINOX is preferred for carefully selected patients, according to country-specific prescription regulations, patient age, clinical conditions, and treatment aim (eg, disease control vs tumor shrinkage).^17^ With the introduction of NALIRIFOX as a new active regimen in this setting,^5^ there is an ongoing debate on how this regimen compares with the very similar FOLFIRINOX.

Our work represents, with all the inevitable limitations, a comparison among these 3 regimens. In terms of activity, our analysis found that the NALIRIFOX and FOLFIRINOX regimens were associated with more overall efficacy than GEM-NABP. However, it should be noted that, compared with most phase 3 studies that used GEM-NABP as a standard-of-care backbone, the outcomes associated with this regimen have clearly improved over time, leading in the most recent studies to results similar to those observed with NALIRIFOX and FOLFIRINOX. This may be due to an improving ability of clinicians over the years to manage this regimen and, therefore, to manage its toxic effects and maintain both dose density and intensity. Thus, considering GEM-NABP as a suboptimal option is not straight forward.

Furthermore, there was no significant difference in OS among patients treated with NALIRIFOX compared with those treated with FOLFIRINOX. Indeed, NALIRIFOX failed to break the symbolic wall of 12 months of median OS, thus questioning the real improvement shown in the NAPOLI 3 trial.^5^ This result is even more relevant considering that NALIRIFOX and FOLFIRINOX are similar in terms of type and dosage of the drugs administered, but with an unfavorable cost-effectiveness ratio. In fact, the mean cost per cycle of liposomal irinotecan has been estimated as more than 100-fold that of irinotecan.

In terms of safety, NALIRIFOX was associated with the most favorable toxicity profile, with a lower incidence of hematological toxic effects and peripheral neuropathy, which often represent the limiting adverse events for the other 2 regimens. This profile might be due also to the different drugs dosing, ie, the lower dose of oxaliplatin may explain the favorable rates of peripheral neuropathy compared with FOLFIRINOX. This tolerable toxicity profile and the high ORR make it an interesting regimen in certain settings, such as neoadjuvant or perioperative therapy, in which maximizing tumor shrinkage and minimizing toxic effects are primary objectives. However, it should be noted that FOLFIRINOX is increasingly used in clinical practice and in clinical trials in the nonmetastatic setting as modified FOLFIRINOX (ie, without fluorouracil bolus and with reduced dosage of irinotecan), which is potentially better tolerated and therefore more easily administered in daily practice as well.

Based on all these considerations, what is the future for the treatment of metastatic PDAC? To date, our data suggest that triplet chemotherapy should be considered in all patients, unless specific contraindications are identified. Among these, careful patient selection should be based on the toxicity profile (eg, avoiding nanoliposomal irinotecan in patients at risk for severe complications in case of grade ≥3 diarrhea; reserving NALIRIFOX for patients for whom significant tumor shrinkage is necessary, given the higher response rate, and in whom peripheral neuropathy could compromise treatment adherence, as in long-course patients with long-course diabetes), age, performance status, allergy to 1 specific drug, *DPYD*^18^ or *UGT1A1*^19^ deficiency, or prior modified FOLFIRINOX treatment in the adjuvant setting within 6 months before recurrence. In such patients, GEM-NABP remains a valid option. Moreover, biomarker-driven treatment selection should be encouraged in the future. Previous research has shown that a tumor’s homologous recombination deficiency is associated with sensitivity to platinum-based chemotherapy.^20,21^ In this light, testing for germline *BRCA1-2* alterations should be routinely performed, as recommended by most guidelines,^22,23^ while there is increasing interest toward the assessment of somatic homologous recombination deficiency, including but not limited to *BRCA1-2* or *PALB2* alterations. Ultimately, our data do not suggest a preference between NALIRIFOX and FOLFIRINOX, which can thus be still considered a valid option to be further explored in its modified version in the metastatic disease setting.

### Limitations

Our work has limitations that should be carefully considered for interpretation of results. First, reconstructed IPD were used, so we were unable to adjust for other pertinent patient-level covariates. Heterogeneity among the populations of the different trials may affect the pooled results. For example, trials testing FOLFIRINOX had an age cap while those studying GEM-NABP (including NAPOLI 3) treated patients older than 76 years, although median age was similar across all studies. Similarly, of the 7 studies assessed,^1,2,5,12,13,14,15^ 3 studies^1,13,15^ did not allow prior adjuvant treatment, while 4 studies^2,5,12,14^ did, with different intervals from adjuvant treatment suspension to start of first-line treatment. Among studies that allowed prior adjuvant treatment, AVENGER500^12^ used the modified FOLFIRINOX regimen in the experimental group, so that patients could not be treated with the same regimen in the adjuvant setting. However, since we included phase 3 RCTs with globally comparable inclusion criteria, the risk of bias due to these limitations should be minimal.

As a further limitation, some response and toxic effects data were available only in a subset of studies. Also, the sample size in the 3 considered groups was unbalanced, with most patients being treated with GEM-NABP. NALIRIFOX has been studied in only 1 phase 3 RCT in this setting, with a median follow up that is currently shorter than that of the studies investigating the other 2 regimens, thus a judgment on its effectiveness may be not conclusive. It will therefore be necessary to wait for longer follow up data to draw conclusions in terms of outcome.

## Conclusions

This systematic review and meta-analysis is the first study, to our knowledge, to report head-to-head comparisons among NALIRIFOX, FOLFIRINOX, and GEM-NABP and may serve as a benchmark for future studies evaluating first-line treatment of metastatic PDAC. These findings may empower a more careful evaluation of these regimens, highlighting the need for careful patient selection and financial toxic effects consideration.

## Notes

Supplement 1. **eFigure 1.** PRISMA Flow Diagram **eFigure 2.** Reconstructed Kaplan-Meier Plots for Progression-Free Survival and Overall Survival According to First-Line Regimen With Follow-Up Censored at the Time of the Shortest Follow-Up Among Included Studies **eFigure 3.** Forest Plots of Progression-Free Survival and Overall Survival in the Network Meta-Analysis **eFigure 4.** Forest Plots of Progression-Free Survival and Overall Survival in Patients Treated With Gemcitabine Plus Nab-Paclitaxel According to the Respective Clinical Trial **eFigure 5.** Best Response and Overall Response Rate According to the Pooled Treatment Arms **eTable 1.** Descriptive Summary of Included Arms of Selected Clinical Trials **eTable 2.** Risk-of-Bias Assessment **eTable 3.** Comparisons to Original Kaplan-Meier Plots and Survival Outcomes **eTable 4.** Survival Analysis and Adjusted Cox Regression Models for Overall Survival and Progression Free Survival **eTable 5.** 6- and 12-Months OS and PFS **eTable 6.** Power Analysis **eTable 7.** Results of Logistic Regression Analysis for G≥3 Toxicities According to Pooled Treatment Regimens

Supplement 2.
Data Sharing Statement

## References

[1] Conroy T, Desseigne F, Ychou M, ; Groupe Tumeurs Digestives of Unicancer; PRODIGE Intergroup. FOLFIRINOX versus gemcitabine for metastatic pancreatic cancer. N Engl J Med. 2011;364(19):1817-1825. doi:10.1056/NEJMoa1011923 21561347

[2] Von Hoff DD, Ervin T, Arena FP, . Increased survival in pancreatic cancer with nab-paclitaxel plus gemcitabine. N Engl J Med. 2013;369(18):1691-1703. doi:10.1056/NEJMoa1304369 24131140 PMC4631139

[3] Pusceddu S, Ghidini M, Torchio M, . Comparative effectiveness of gemcitabine plus nab-paclitaxel and FOLFIRINOX in the first-line setting of metastatic pancreatic cancer: a systematic review and meta-analysis. Cancers (Basel). 2019;11(4):484. doi:10.3390/cancers11040484 30959763 PMC6520876

[4] Takumoto Y, Sasahara Y, Narimatsu H, Akazawa M. Comparative outcomes of first-line chemotherapy for metastatic pancreatic cancer among the regimens used in Japan: a systematic review and network meta-analysis. JAMA Netw Open. 2022;5(1):e2145515. doi:10.1001/jamanetworkopen.2021.45515 35099549 PMC8804927

[5] Wainberg ZA, Melisi D, Macarulla T, . NALIRIFOX versus nab-paclitaxel and gemcitabine in treatment-naive patients with metastatic pancreatic ductal adenocarcinoma (NAPOLI 3): a randomised, open-label, phase 3 trial. Lancet. 2023;402(10409):1272-1281. doi:10.1016/S0140-6736(23)01366-1 37708904 PMC11664154

[6] Sterne JAC, Savović J, Page MJ, . ROB 2: a revised tool for assessing risk of bias in randomised trials. BMJ. 2019;366:l4898. doi:10.1136/bmj.l4898 31462531

[7] Guyot P, Ades AE, Ouwens MJ, Welton NJ. Enhanced secondary analysis of survival data: reconstructing the data from published Kaplan-Meier survival curves. BMC Med Res Methodol. 2012;12:9. doi:10.1186/1471-2288-12-9 22297116 PMC3313891

[8] Liu N, Zhou Y, Lee JJ. IPDfromKM: reconstruct individual patient data from published Kaplan-Meier survival curves. BMC Med Res Methodol. 2021;21(1):111. doi:10.1186/s12874-021-01308-8 34074267 PMC8168323

[9] Zhao JJ, Yap DWT, Chan YH, . Low programmed death-ligand 1-expressing subgroup outcomes of first-line immune checkpoint inhibitors in gastric or esophageal adenocarcinoma. J Clin Oncol. 2022;40(4):392-402. doi:10.1200/JCO.21.01862 34860570

[10] Pietrantonio F, Miceli R, Raimondi A, . Individual patient data meta-analysis of the value of microsatellite instability as a biomarker in gastric cancer. J Clin Oncol. 2019;37(35):3392-3400. doi:10.1200/JCO.19.01124 31513484

[11] Raimondi A, Nichetti F, Stahler A, . Optimal maintenance strategy following FOLFOX plus anti-EGFR induction therapy in patients with RAS wild type metastatic colorectal cancer: an individual patient data pooled analysis of randomised clinical trials. Eur J Cancer. 2023;190:112945. doi:10.1016/j.ejca.2023.112945 37441940

[12] Philip PA, Buyse ME, Alistar AT, . Avenger 500, a phase III open-label randomized trial of the combination of CPI-613 with modified FOLFIRINOX (mFFX) versus FOLFIRINOX (FFX) in patients with metastatic adenocarcinoma of the pancreas. J Clin Oncol. 2019;37(4)(suppl):TPS479. doi:10.1200/JCO.2019.37.4_suppl.TPS479

[13] Bekaii-Saab T, Okusaka T, Goldstein D, . Napabucasin plus nab-paclitaxel with gemcitabine versus nab-paclitaxel with gemcitabine in previously untreated metastatic pancreatic adenocarcinoma: an adaptive multicentre, randomised, open-label, phase 3, superiority trial. EClinicalMedicine. 2023;58:101897. doi:10.1016/j.eclinm.2023.101897 36969338 PMC10036520

[14] Van Cutsem E, Tempero MA, Sigal D, ; HALO 109-301 Investigators. Randomized phase III trial of pegvorhyaluronidase alfa with nab-paclitaxel plus gemcitabine for patients with hyaluronan-high metastatic pancreatic adenocarcinoma. J Clin Oncol. 2020;38(27):3185-3194. doi:10.1200/JCO.20.00590 32706635 PMC7499614

[15] Tempero M, Oh DY, Tabernero J, . Ibrutinib in combination with nab-paclitaxel and gemcitabine for first-line treatment of patients with metastatic pancreatic adenocarcinoma: phase III RESOLVE study. Ann Oncol. 2021;32(5):600-608. doi:10.1016/j.annonc.2021.01.070 33539945

[16] Fu Q, Chen Y, Huang D, . Randomized phase III study of sintilimab in combination with modified folfrinox versus folfrinox alone in patients with metastatic and recurrent pancreatic cancer in China: The CISPD3 trial. J Clin Oncol. 2022;40(4)(suppl):560. doi:10.1200/JCO.2022.40.4_suppl.560

[17] Reni M, Giommoni E, Bergamo F, ; GARIBALDI Study Group. Guideline application in real world: multi-institutional based survey of adjuvant and first-line pancreatic ductal adenocarcinoma treatment in Italy. primary analysis of the GARIBALDI survey. ESMO Open. 2023;8(1):100777. doi:10.1016/j.esmoop.2022.100777 36731325 PMC10024128

[18] Innocenti F, Mills SC, Sanoff H, Ciccolini J, Lenz HJ, Milano G. All you need to know about *DPYD* genetic testing for patients treated with fluorouracil and capecitabine: a practitioner-friendly guide. JCO Oncol Pract. 2020;16(12):793-798. doi:10.1200/OP.20.00553 33197222 PMC8462561

[19] Karas S, Innocenti F. All you need to know about *UGT1A1* genetic testing for patients treated with irinotecan: a practitioner-friendly guide. JCO Oncol Pract. 2022;18(4):270-277. doi:10.1200/OP.21.00624 34860573 PMC9014426

[20] Park W, Chen J, Chou JF, . Genomic methods identify homologous recombination deficiency in pancreas adenocarcinoma and optimize treatment selection. Clin Cancer Res. 2020;26(13):3239-3247. doi:10.1158/1078-0432.CCR-20-0418 32444418 PMC7380542

[21] Stossel C, Raitses-Gurevich M, Atias D, . Spectrum of response to platinum and PARP inhibitors in germline BRCA-associated pancreatic cancer in the clinical and preclinical setting. Cancer Discov. 2023;13(8):1826-1843. doi:10.1158/2159-8290.CD-22-0412 37449843 PMC10401074

[22] Sohal DPS, Kennedy EB, Cinar P, . Metastatic pancreatic cancer: ASCO guideline update. J Clin Oncol. 2020;38(27):3217-3230. doi:10.1200/JCO.20.01364 32755482 PMC12974607

[23] Ducreux M, Cuhna AS, Caramella C, ; ESMO Guidelines Committee. Cancer of the pancreas: ESMO clinical practice guidelines for diagnosis, treatment and follow-up. Ann Oncol. 2015;26(suppl 5):v56-v68. doi:10.1093/annonc/mdv295 26314780

