# Supplementary material for: NALIRIFOX, FOLFIRINOX, and Gemcitabine With Nab-Paclitaxel as First-Line Chemotherapy for Metastatic Pancreatic Cancer: A Systematic Review and Meta-Analysis
Source: JAMA Netw Open. 2024 Jan 8;7(1):e2350756. doi: 10.1001/jamanetworkopen.2023.50756 (PMC10774994; doi:10.1001/jamanetworkopen.2023.50756)
Supplement: Supplement 1. — eFigure 1. PRISMA Flow Diagram eFigure 2. Reconstructed Kaplan-Meier Plots for Progression-Free Survival and Overall Survival According to First-Line Regimen With Follow-Up Censored at the Time of the Shortest Follow-Up Among Included Studies eFigure 3. Forest Plots of Progression-Free Survival and Overall Survival in the Network Meta-Analysis eFigure 4. Forest Plots of Progression-Free Survival and Overall Survival in Patients Treated With Gemcitabine Plus Nab-Paclitaxel According to the Respective Clinical Trial eFigure 5. Best Response and Overall Response Rate According to the Pooled Treatment Arms eTable 1. Descriptive Summary of Included Arms of Selected Clinical Trials eTable 2. Risk-of-Bias Assessment eTable 3. Comparisons to Original Kaplan-Meier Plots and Survival Outcomes eTable 4. Survival Analysis and Adjusted Cox Regression Models for Overall Survival and Progression Free Survival eTable 5. 6- and 12-Months OS and PFS eTable 6. Power Analysis eTable 7. Results of Logistic Regression Analysis for G≥3 Toxicities According to Pooled Treatment Regimens [file jamanetwopen-e2350756-s001.pdf]

Supplemental Online Content

Nichetti F, Rota S, Ambrosini P, et al. NALIRIFOX vs FOLFIRINOX vs gemcitabine with nab-paclitaxel as first-line chemotherapy for metastatic pancreatic cancer: a systematic review and meta-analysis. *JAMA Netw Open*. 2024;7(1):e2350756. doi:10.1001/jamanetworkopen.2023.50756

eFigure 1. PRISMA Flow Diagram
eFigure 2. Reconstructed Kaplan-Meier Plots for Progression-Free Survival and Overall Survival According to First-Line Regimen With Follow-Up Censored at the Time of the Shortest Follow-Up Among Included Studies
eFigure 3. Forest Plots of Progression-Free Survival and Overall Survival in the Network Meta-Analysis
eFigure 4. Forest Plots of Progression-Free Survival and Overall Survival in Patients Treated With Gemcitabine Plus Nab-Paclitaxel According to the Respective Clinical Trial
eFigure 5. Best Response and Overall Response Rate According to the Pooled Treatment Arms
eTable 1. Descriptive Summary of Included Arms of Selected Clinical Trials
eTable 2. Risk-of-Bias Assessment
eTable 3. Comparisons to Original Kaplan-Meier Plots and Survival Outcomes
eTable 4. Survival Analysis and Adjusted Cox Regression Models for Overall Survival and Progression Free Survival
eTable 5. 6- and 12-Months OS and PFS
eTable 6. Power Analysis
eTable 7. Results of Logistic Regression Analysis for G≥3 Toxicities According to Pooled Treatment Regimens

This supplemental material has been provided by the authors to give readers additional information about their work.

© 2023 Nichetti F et al. *JAMA Network Open.*

Supplementary Methods:

1. Study selection guidelines

Date of search: September 12th, 2023

On **Pubmed** (http://www.ncbi.nlm.nih.gov/pubmed), the following query string was applied: *'((2011/01/01:2023/06/01[Date - Publication])) AND ((clinicaltrial[Filter])))) AND (metastatic pancreatic cancer[MeSH Terms])'*.

On **Embase** (https://www.embase.com/search/), the following query string was applied: *`pancreatic:ti,ab,kw AND cancer:ti,ab,kw AND 'first line':ti,ab,kw AND 'phase 3':ti,ab,kw AND (2011:py OR 2012:py OR 2013:py OR 2015:py OR 2016:py OR 2017:py OR 2018:py OR 2019:py OR 2020:py OR 2021:py OR 2022:py OR 2023:py) AND ('adenocarcinoma'/dm OR 'pancreas adenocarcinoma'/dm OR 'pancreas cancer'/dm OR 'pancreas metastasis'/dm) AND ('Article'/it OR 'Conference Abstract'/it)`.*

On **Scopus** (https://www.scopus.com/search/), *`( TITLE-ABS-KEY ( pancreatic AND cancer ) AND TITLE-ABS-KEY ( first-line ) AND TITLE-ABS-KEY ( phase AND iii ) OR TITLE-ABS-KEY ( phase 3 ) ) AND PUBYEAR > 2010`.*

On **ESMO** meetings resource (https://oncologypro.esmo.org/), the keywords `*phase 3*` and `*phase III*` were searched after the following filters were applied:

- Pancreatic adenocarcinoma as **Tumor Site**;
- Meeting Resources as **Sections**.

© 2023 Nichetti F et al. *JAMA Network Open.*

On **ASCO** meetings resource (https://meetings.asco.org/abstracts-presentations/), the keywords `*phase 3*` and `*phase III*` were searched after the following filters were applied:

- ASCO annual meeting and Gastrointestinal Cancers Symposium as **Meeting;**
- Cancers Gastrointestinal cancers Pancreatic cancer as **Topic.**

All results were downloaded, checked for overlap between the different sources, and then hierarchically removed according to the following pre-specified criteria:

1) First, the study was removed if not concerning pancreatic cancer;
2) if yes, the study was removed if it was not a phase III clinical trial;
3) if yes, the study was removed if not concerning first-line treatment;
4) if yes, the study was removed if including Gem-NabP, FOLFIRINOX or NALIRIFOX, planned at standard dose density and intensity, at least as one treatment arm;
5) if yes, the study was removed if concerning first-line treatment of locally advanced, unresectable but not metastatic pancreatic cancer;
6) if yes, the study was removed if PFS and OS Kaplan Meier plots with number-at-risk tables were not available as main or supplementary figures.

© 2023 Nichetti F et al. *JAMA Network Open.*

2. Statistical methods for individual patient data reconstruction and Cox regression analysis

The IPDfromKM was adopted to reconstruct time-to-event outcomes. IPDfromKM is a graphical algorithm that, starting from point coordinates of each step (i.e. censoring mark or event) and from at-risk-tables in a KM plot, allows to reconstruct individual patient survival data (months and censoring status). Reconstruction accuracy was assessed by a) comparing median OS and PFS (together with 95% confidence intervals, CIs) and hazard ratios (HRs, together with 95% CIs) to original publications; a +/-0.4 months (i.e. < 2 weeks) or +/-0.2 HR difference from the original papers was tolerated as acceptable in reconstruction; b) by inspecting the visual shape of plots and number-at-risk tables of extracted data compared with originals; c) Kolmogorov-Smirnov test and evaluation of root-mean-square error (RMSE) between estimated and read-in survival probabilities.

After applying this procedure to each KM plot of the included trials, patients from different trials treated with the same regimen were merged in unique groups, keeping the annotation of each patient's original trial.

Then, the impact of each arm was investigated with Cox regression models, keeping the NALIRIFOX group as a reference, and reported as hazard ratios (HRs) with 95% confidence intervals (CI). In each Cox model, the clinical trial of origin (e.g. NAPOLI-3, HALO, etc) was included as a random variable (i.e. 1|trial in the R language, using the coxme package) to take into account the potential difference due to patient's belonging to a specific clinical trial and the size of each trial. Further details can be found in the original IPDfromKM paper and in the coxme package manual. Results were validated using a frequentist network meta-analysis using the netmeta R package.

3. List of R packages used for the analysis

Analyses were conducted using the R Statistical language (version 4.2.2; R Core Team, 2022) on macOS Catalina 10.15.7, using the packages

- gridExtra (version 2.3; Auguie B, 2017),
- easyPubMed (version 2.13; Fantini D, 2019),

© 2023 Nichetti F et al. *JAMA Network Open.*

- effectsize (version 0.8.3; Ben-Shachar MS et al., 2020),
- epiDisplay (version 3.5.0.2; Chongsuvivatwong V, 2022),
- ggsignif (version 0.6.4; Constantin A, Patil I, 2021),
- janitor (version 2.2.0; Firke S, 2023),
- TrialSize (version 1.4; G.Zhang EZ;VQW;SC;H, 2020),
- Prodlim (version 2023.3.31; Gerds TA, 2023),
- lubridate (version 1.9.2; Grolemund G, Wickham H, 2011),
- Hmisc (version 5.1.0; Harrell Jr F, 2023),
- rms (version 6.7.0; Harrell Jr FE, 2023),
- gt (version 0.9.0; Iannone R et al., 2023),
- readbitmap (version 0.1.5; Jefferis G, 2018),
- ehahelper (version 0.3.9999; Junkka J, 2020),
- ggpubr (version 0.6.0; Kassambara A, 2023),
- survminer (version 0.4.9; Kassambara A et al., 2021),
- IPDfromKM (version 0.1.10; Liu N, LeeJ, 2020),
- parameters (version 0.21.1; Lüdecke D et al., 2020),
- performance (version 0.10.4; Lüdecke D et al., 2021),
- easystats (version 0.6.0; Lüdecke D et al., 2022),
- see (version0.8.0; Lüdecke D et al., 2021),
- insight (version 0.19.2; Lüdecke D et al., 2019),
- bayestestR (version 0.13.1; Makowski D et al., 2019),
- modelbased (version 0.8.6; Makowski D et al., 2020),
- report (version 0.5.7; Makowski D et al., 2023),
- correlation (version 0.8.4; MakowskiD et al., 2022),
- tibble (version 3.2.1; Müller K, Wickham H, 2023),
- datawizard (version 0.7.1; Patil I et al., 2022),
- powerSurvEpi (version 0.1.3; Qiu W et al., 2021),
- foreign (version 0.8.84; R Core Team, 2022),

© 2023 Nichetti F et al. *JAMA Network Open.*

- broom (version 1.0.5; Robinson D et al., 2023),
- hrbrthemes (version 0.8.0; Rudis B, 2020),
- gtsummary (version 1.7.1; Sjoberg D et al., 2021),
- bdsmatrix (version 1.3.6; Therneau T, 2022),
- survival (version 3.5.5; Therneau T, 2023),
- coxme (version 2.2.18.1; Therneau TM, 2022),
- MASS (version 7.3.60; Venables WN, Ripley BD,2002),
- nnet (version 7.3.19; Venables WN, Ripley BD, 2002),
- ggplot2 (version 3.4.2; WickhamH, 2016),
- stringr (version 1.5.0; Wickham H, 2022),
- forcats (version 1.0.0; Wickham H, 2023),
- tidyverse (version 2.0.0; Wickham H et al., 2019),
- dplyr (version 1.1.2; Wickham H et al.,2023),
- purrr (version 1.0.1; Wickham H, Henry L, 2023),
- readr (version 2.1.4; Wickham H et al., 2023),
- tidyr (version 1.3.0; Wickham H et al., 2023),
- broom.mixed (version 0.2.9.4; Bolker B, Robinson D, 2022),
- lme4 (version 1.1.33; Bates D et al., 2015),
- netmeta (version 2.8.2; Balduzzi S et al., 2023),
- meta (version 6.5.0; Balduzzi S et al., 2019).

© 2023 Nichetti F et al. *JAMA Network Open.*

Supplementary Tables:

Supplementary Table 1: Descriptive summary of included arms of selected clinical trials.

Characteristic	Detail	NAPOLI-3 (NALIRIFOX)	NAPOLI-3 (Gem-NabP)	ACCORD 11 (FOLFIRINOX)	MPACT (Gem-NabP)	HALO (Gem-NabP)	RESOLVE (Gem-NabP)	AVENGER500 (FOLFIRINOX)	CanStem111P (Gem-NabP)
**Sex**	Female	180 (46.7%)	157 (40.6%)	65 (38.0%)	186 (43.2%)	85 (51.5%)	92 (43%)	102 (39%)	263 (46.2%)
**Age**	Median (min - max)	64 (20 - 85)	65 (36 - 82)	61 (25 - 76)	62 (27 - 86)	62.3 (\ )	64 (32 - 85)	63 (29 - 75)	64 (57 - 70)
**ECOG PS**	0	160 (41.8%)	168 (43.4%)	64 (37.4%)	69 (16.1%)	79 (47.9%)	46 (22.0%)	126 (48.0%)	255 (44.8%)
	1	223 (58.0%)	219 (56.6%)	106 (61.9%)	328 (76.5%)	86 (52.1%)	154 (72.0%)*	136 (52.0%)	314 (55.2%)
**CA 19-9**		≥37*UNL in 83.8%	≥37*UNL in 81.7%	≥59*UNL in 41.5%	≥59*UNL in 51.9%	21.78 U/ml (mean)	\	≥59*UNL in 42%	≥59*UNL in 44.6%
**Liver metastases**	Yes	307 (80.2%)	311 (80.4%)	149 (87.6%)	365 (84.7%)	124 (75.2%)	172 (81%)	199 (76.0%)	446 (78.4%)
**Median OS**	months (95% confidence interval)	11.1 (10.0 - 12.1)	9.2 (8.3 - 10.6)	11.1 (9.0 - 13.1)	8.5 (7.9 - 9.5)	11.5 (9.0 - 12.5)	10.8 (8.9 - 11.7)	11.7 (10.1 - 13.2)	11.7 (10.7 - 12.7)
**Median PFS**	months (95% confidence interval)	7.4 (6.0 - 7.7)	5.6 (5.3 - 5.8)	6.4 (5.5 - 7.2)	5.5 (4.5 - 5.9)	7.1 (4.8 - 8.3)	6.01 (5.5 - 7.2)	8.0 (7.2 - 11.1)	6.1 (5.6 - 7.1)
**Best Response**	CR	1 (0.3%)	1 (0.3%)	1 (0.6%)	1 (0.2%)	1 (0.6%)	3 (1.4%)	\	6 (1.1%)
	PR	139 (41.5%)	159 (35.9%)	53 (31.0%)	98 (22.7%)	59 (35.7%)	87 (41.0%)	\	234 (41.9%)
	SD	101 (25.8%)	99 (26.0%)	66 (38.6%)	118 (27.4%)	54 (32.7%)	\	\	185 (33.1%)
	PD	56 (9.9%)	38 (14.5%)	26 (15.2%)	86 (19.9%)	22 (13.3%)	\	\	62 (11.1%)
	Not Evaluable	90 (22.5%)	86 (23.3%)	25 (14.6%)	128 (29.7%)	29 (17.6%)	\	\	72 (12.9%)
**ORR**	yes	41.8%	36.2%	31.6%	23.0%	36.3%	42.3%	34.0%	42.9%

© 2023 Nichetti F et al. *JAMA Network Open.*

	Not Evaluable	22.5%	23.3%	14.6%	29.7%	17.6%	\	\	12.8%
**Grade ≥3 toxicity**	Anemia	10.5%	17.8%	7.8%	13.1%	23.9%	16.9%	13.6%	19.7%
	Neutrophil count decreased	23.8%	38.0%	45.7%	37.8%	53.5%	34.9%	20.4%	22.5%
	Febrile Neutropenia	2.4%	2.4%	5.4%	3.3%	6.8%	1.4%	9.4%	6.3%
	Platelet count decreased	1.6%	6.1%	9.1%	12.8%	16.0%	9.9%	13.6%	\
	Diarrhea	20.3%	4.5%	12.7%	5.7%	\	9.0%	19.6%	4.9%
	Peripheal neuropathy	8.7%	7.9%	9.0%	16.6%	\	7.5%	\	\

* 13 (6%) of patients in RESOLVE had Karnofsky = 70 (i.e. ECOG PS 2).
Abbreviations: CA 19-9: carbohydrate antigen 19.9; CR: complete response; ECOG: eastern ocooperative oncology group; Gem-NabP: gemcitabine plus Nab-Paclitaxel; NALIRIFOX: 5-fluorouracil, leucovorin, liposomal irinotecan and oxaliplatin; ORR: overall response rate; OS: overall survival; PFS: progression free survival; PD: progressive disease; PR: partial response; SD: stable disease; UNL: upper normal limit.

© 2023 Nichetti F et al. *JAMA Network Open.*

Supplementary Table 2: Risk-of-Bias assessment

	D1	D2	D3	D4	D5	OVERALL
NAPOLI-3	✓	✓	✓	✓	✓	✓
ACCORD11	✓	✓	✓	✓	✓	✓
MPACT	✓	✓	✓	✓	✓	✓
HALO	✓	✓	✓	✓	✓	✓
AVENGER500	✓	✓	✓	✓	✓	✓
RESOLVE	✓	✓	✓	✓	✓	✓
CanStem111P	✓	✓	✓	✓	✓	✓

© 2023 Nichetti F et al. *JAMA Network Open.*

DOMAINS:
D1: Bias from the randomization process
D2: Bias due to deviation from the intended intervention
D3: Bias due to the missing of outcome data
D4: Bias in measurement of the outcome
D5: Bias in selection of the reported result
OVERALL: Overall Risk of Bias

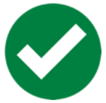 = low risk of bias

© 2023 Nichetti F et al. *JAMA Network Open.*

Supplementary Table 3: Comparisons to original Kaplan Meier plots and survival outcomes.

Trial, outcome, regimen	Original	Reconstructed Kaplan Meier plots

© 2023 Nichetti F et al. *JAMA Network Open.*

NAPOLI-3<br><br>PFS	The original figure may be found in the primary trial manuscript .<br>Wainberg ZA, Melisi D, Macarulla T, et al. NALIRIFOX versus nab-paclitaxel and gemcitabine in treatment-naive patients with metastatic pancreatic ductal adenocarcinoma (NAPOLI 3): a randomised, open-label, phase 3 trial , Lancet. 2023 Oct 7;402(10409):1272-1281. doi: 10.1016/S0140-6736(23)01366-1. PMID: 37708904<br><br>Figure 2B	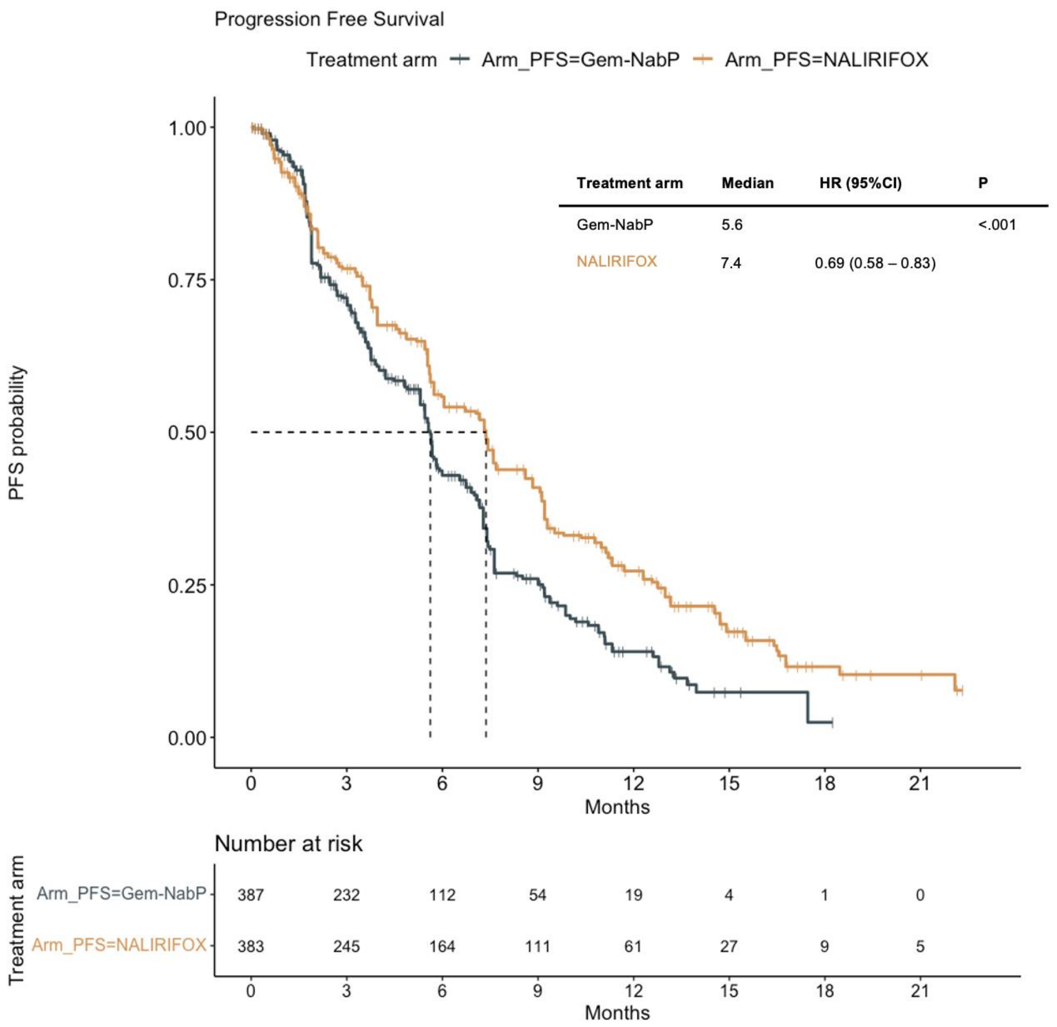

© 2023 Nichetti F et al. *JAMA Network Open.*

NAPOLI-3<br><br>OS	The original figure may be found in the primary trial manuscript .<br>Wainberg ZA, Melisi D, Macarulla T, et al. NALIRIFOX versus nab-paclitaxel and gemcitabine in treatment-naive patients with metastatic pancreatic ductal adenocarcinoma (NAPOLI 3): a randomised, open-label, phase 3 trial, Lancet. 2023 Oct 7;402(10409):1272-1281. doi: 10.1016/S0140-6736(23)01366-1. PMID: 37708904<br><br>Figure 2A	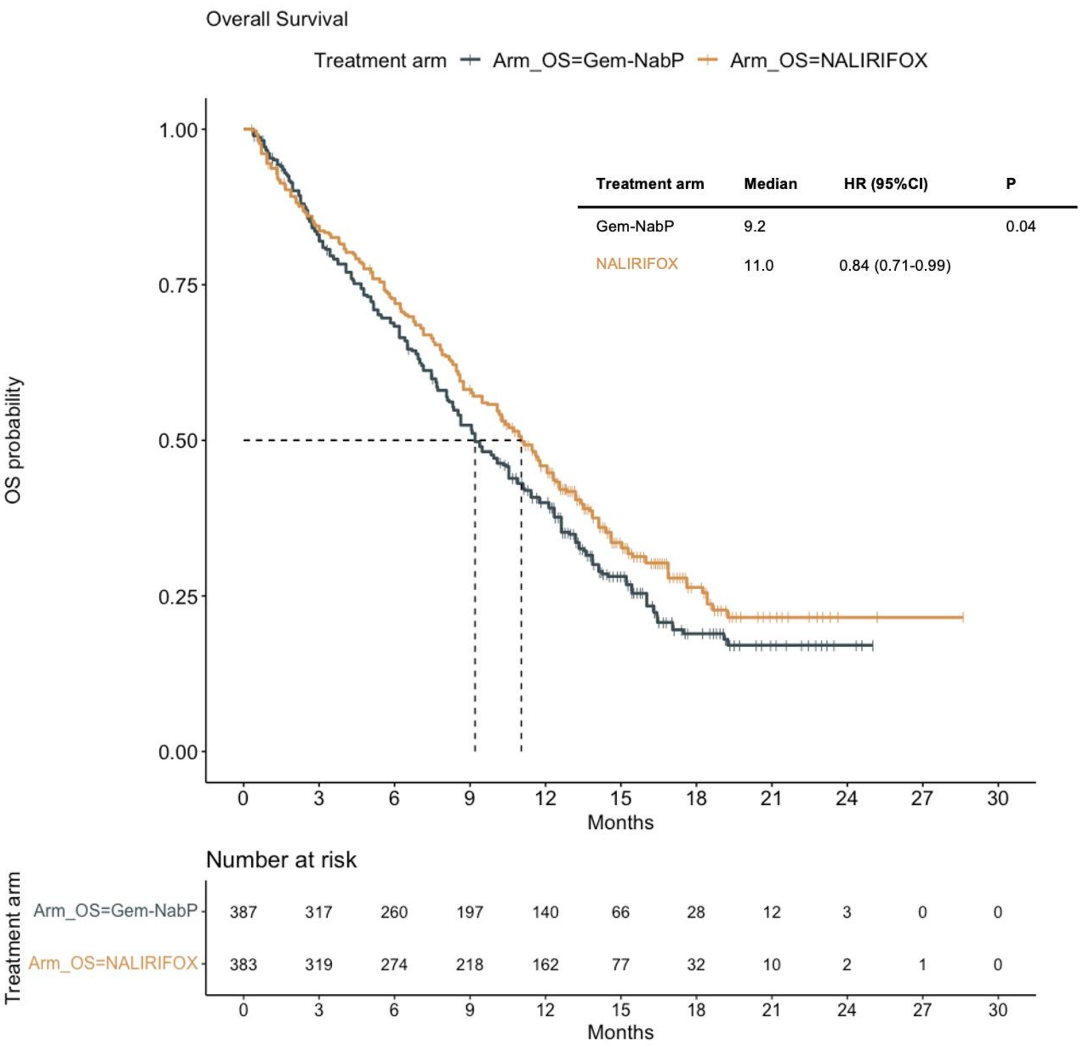

© 2023 Nichetti F et al. *JAMA Network Open.*

ACCORD11<br><br>PFS	The original figure may be found in the primary trial manuscript.<br>Conroy T, Desseigne F, Ychou M, et Al. FOLFIRINOX versus gemcitabine for metastatic pancreatic cancer.<br>N Engl J Med. 2011 May 12;364(19):1817-25. doi: 10.1056/NEJMoa1011923. PMID: 21561347.<br><br>Figure 1B	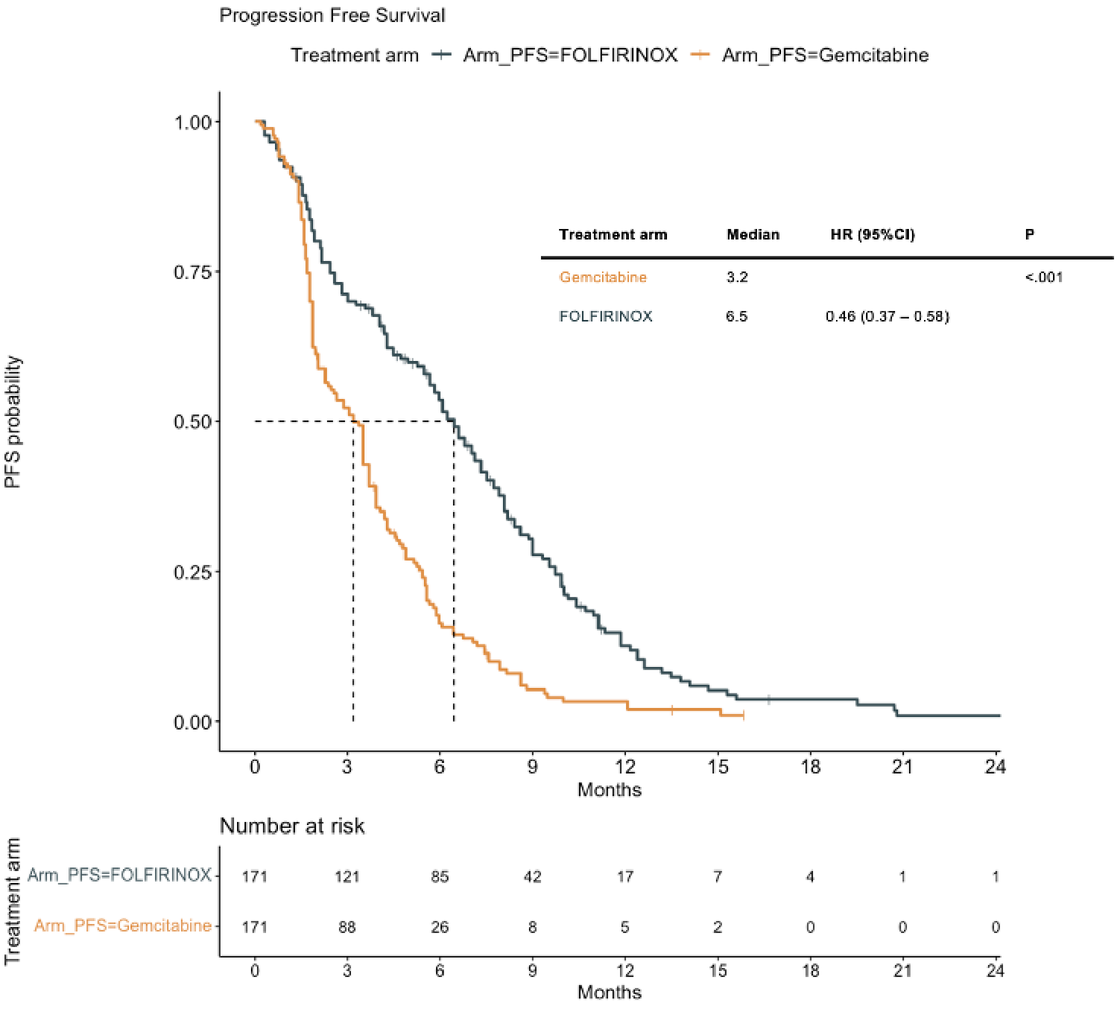

© 2023 Nichetti F et al. *JAMA Network Open.*

ACCORD11<br><br>OS	The original figure may be found in the primary trial manuscript.<br>Conroy T, Desseigne F, Ychou M, et Al. FOLFIRINOX versus gemcitabine for metastatic pancreatic cancer.<br>N Engl J Med. 2011 May 12;364(19):1817-25. doi: 10.1056/NEJMoa1011923. PMID: 21561347.<br><br>Figure 1A	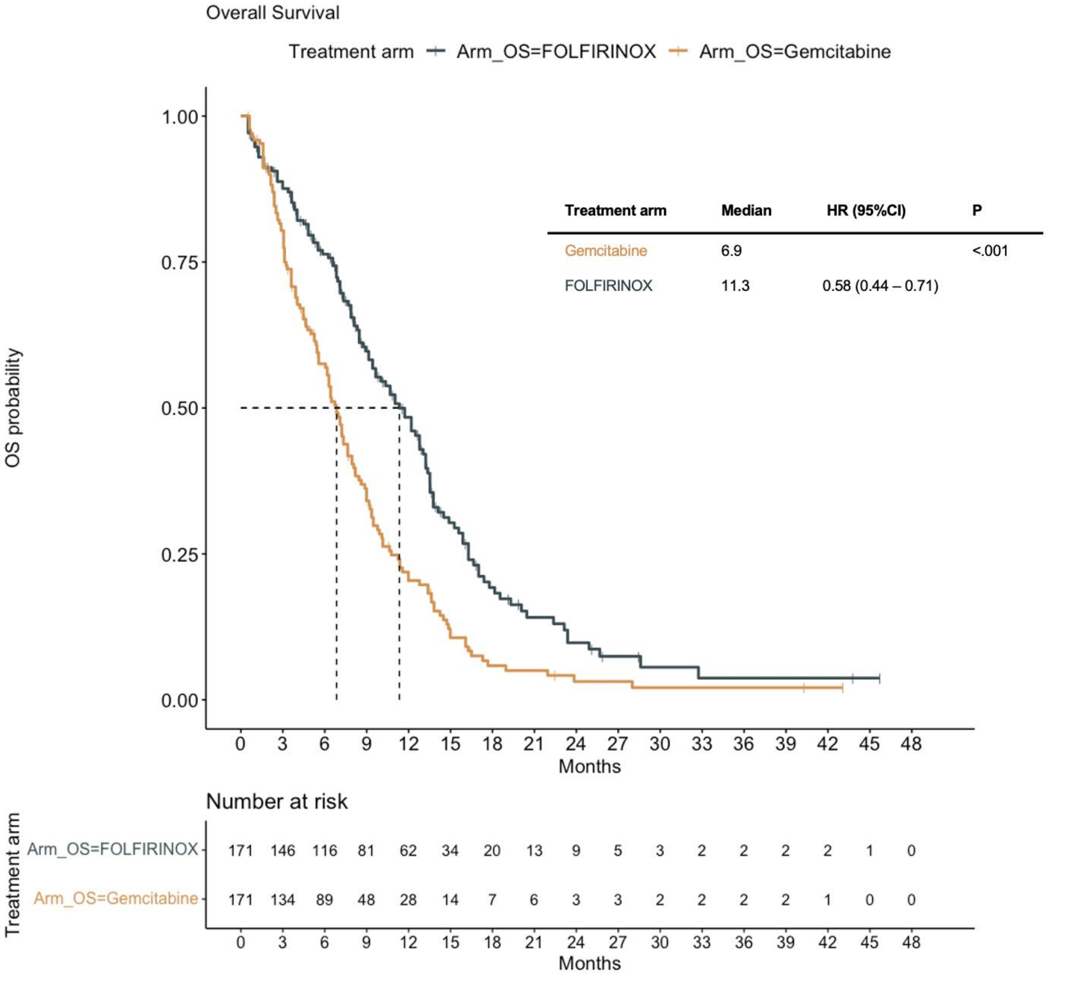

© 2023 Nichetti F et al. *JAMA Network Open.*

MPACT<br><br>PFS	The original figure may be found in the primary trial manuscript.<br>Von Hoff DD, Ervin T, Arena FP, er Al. Increased survival in pancreatic cancer with nab-paclitaxel plus gemcitabine. N Engl J Med. 2013 Oct 31;369(18):1691-703. doi: 10.1056/NEJMoa1304369. Epub 2013 Oct 16. PMID: 24131140; PMCID: PMC4631139.<br><br>Figure 1B	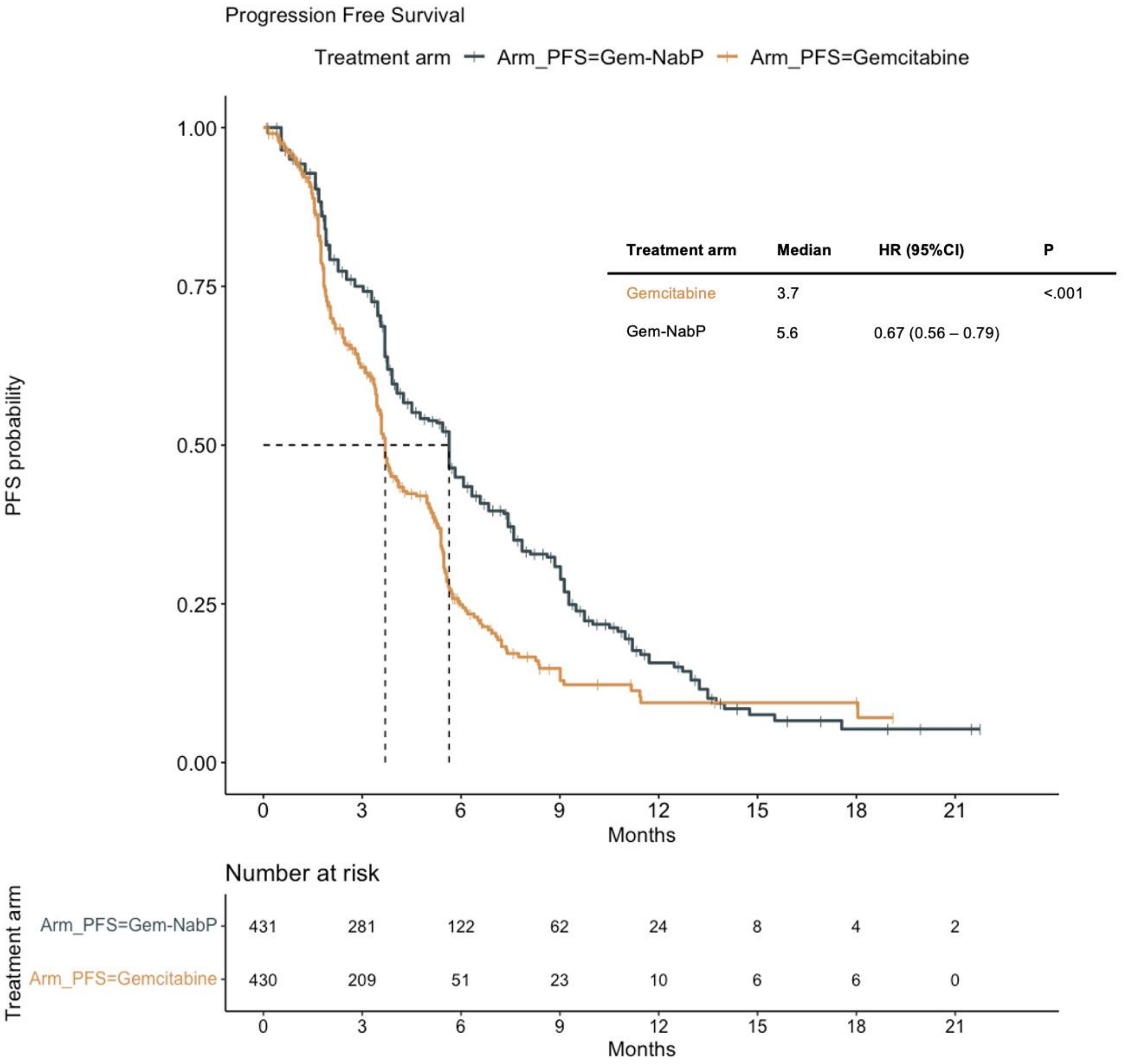

© 2023 Nichetti F et al. *JAMA Network Open.*

MPACT<br><br>OS	The original figure may be found in the primary trial manuscript.<br>Von Hoff DD, Ervin T, Arena FP, er Al. Increased survival in pancreatic cancer with nab-paclitaxel plus gemcitabine. N Engl J Med. 2013 Oct 31;369(18):1691-703. doi: 10.1056/NEJMoa1304369. Epub 2013 Oct 16. PMID: 24131140; PMCID: PMC4631139.<br><br>Figure 1A	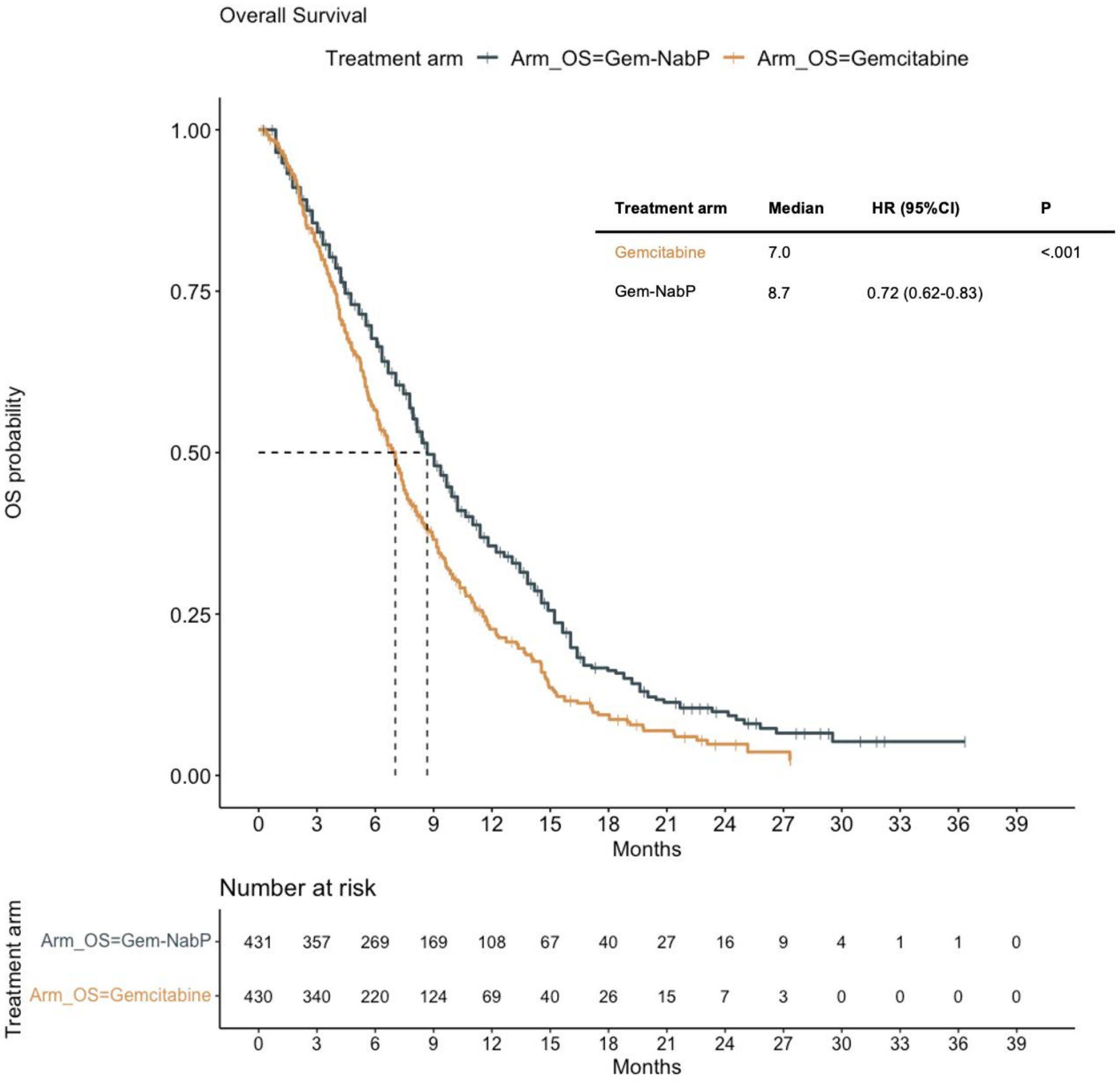

© 2023 Nichetti F et al. *JAMA Network Open.*

HALO<br><br>PFS	The original figure may be found in the primary trial manuscript.<br>Van Cutsem E, Tempero MA, Sigal D, et Al. Randomized Phase III Trial of Pegvorhyaluronidase Alfa With Nab-Paclitaxel Plus Gemcitabine for Patients With Hyaluronan-High Metastatic Pancreatic Adenocarcinoma. J Clin Oncol. 2020 Sep 20;38(27):3185-3194. doi: 10.1200/JCO.20.00590. Epub 2020 Jul 24. PMID: 32706635; PMCID: PMC7499614.<br><br>Figure 2B	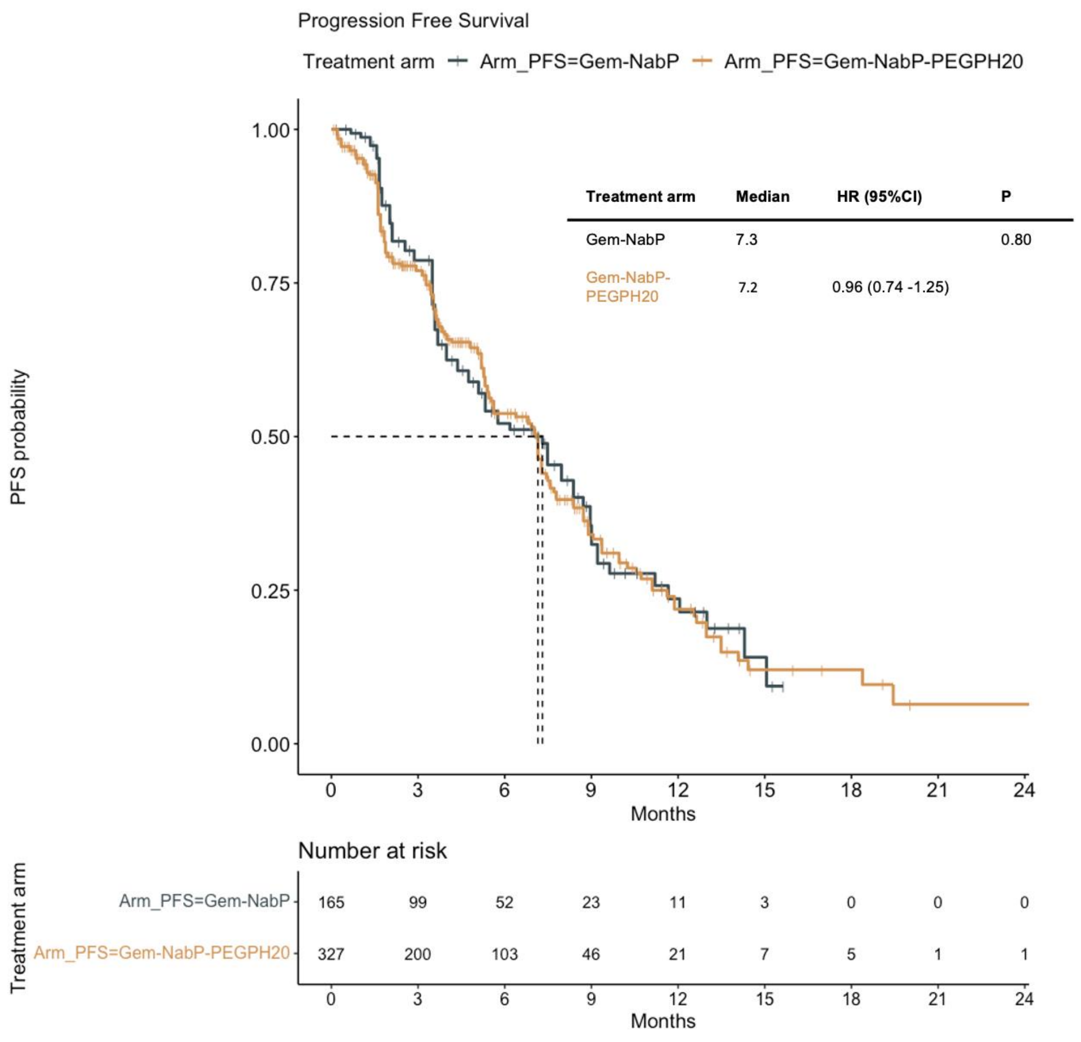

© 2023 Nichetti F et al. *JAMA Network Open.*

HALO<br><br>OS	The original figure may be found in the primary trial manuscript.<br>Van Cutsem E, Tempero MA, Sigal D, et Al. Randomized Phase III Trial of Pegvorhyaluronidase Alfa With Nab-Paclitaxel Plus Gemcitabine for Patients With Hyaluronan-High Metastatic Pancreatic Adenocarcinoma. J Clin Oncol. 2020 Sep 20;38(27):3185-3194. doi: 10.1200/JCO.20.00590. Epub 2020 Jul 24. PMID: 32706635; PMCID: PMC7499614.<br><br>Figure 2A	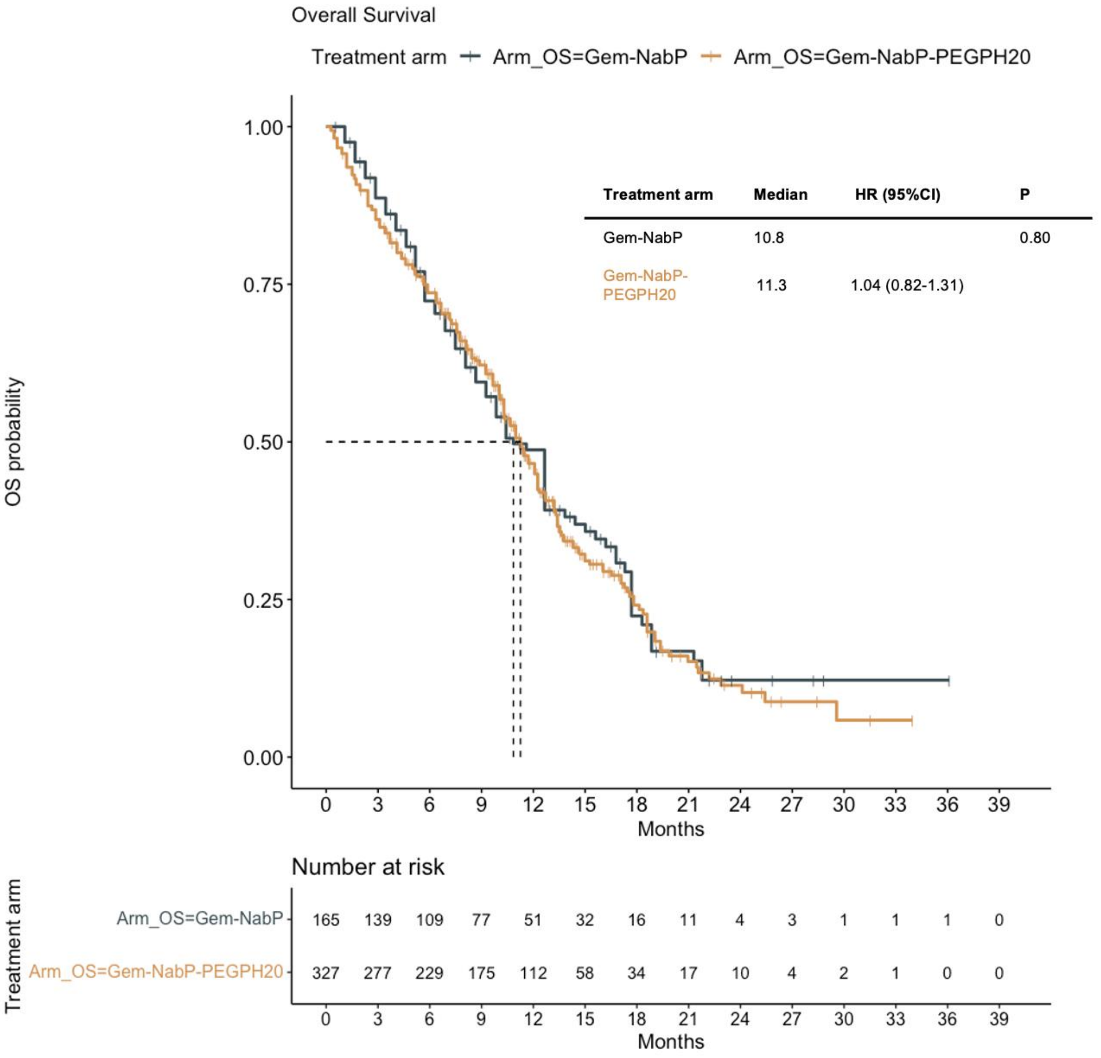

© 2023 Nichetti F et al. *JAMA Network Open.*

AVENGER500<br><br>PFS	The original figure may be found in the trial presented poster.<br>Philip PA, Buyse ME, Alistar AT, et al: Avenger 500, a phase III open-label randomized trial of the combination of CPI-613 with modified FOLFIRINOX (mFFX) versus FOLFIRINOX (FFX) in patients with metastatic adenocarcinoma of the pancreas. Journal of Clinical Oncology 37:TPS479-TPS479, 2019<br><br>Figure 1	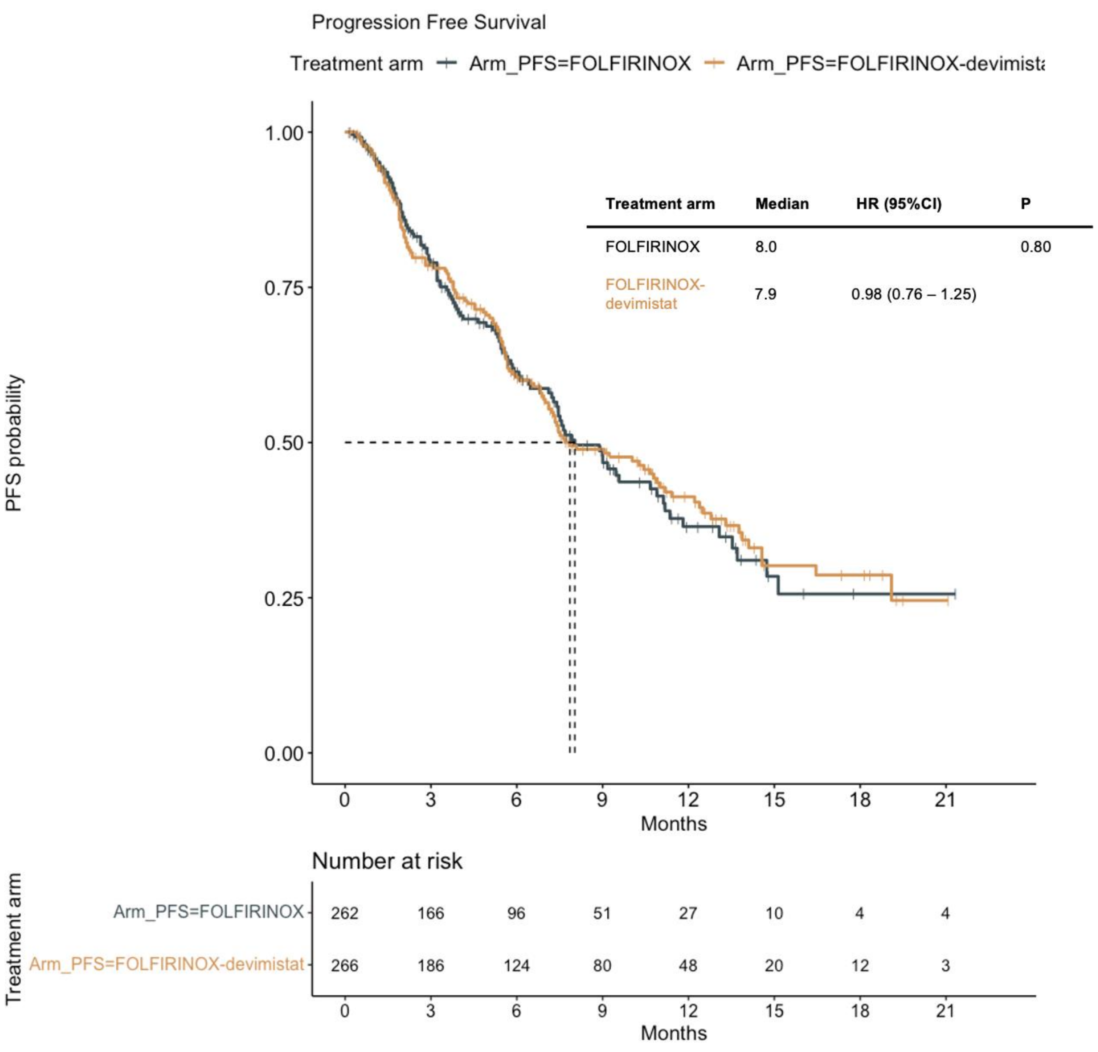

© 2023 Nichetti F et al. *JAMA Network Open.*

AVENGER500<br><br>OS	The original figure may be found in the trial presented poster. Philip PA, Buyse ME, Alistar AT, et al: Avenger 500, a phase III open-label randomized trial of the combination of CPI-613 with modified FOLFIRINOX (mFFX) versus FOLFIRINOX (FFX) in patients with metastatic adenocarcinoma of the pancreas. Journal of Clinical Oncology 37:TPS479-TPS479, 2019<br><br>Figure 1	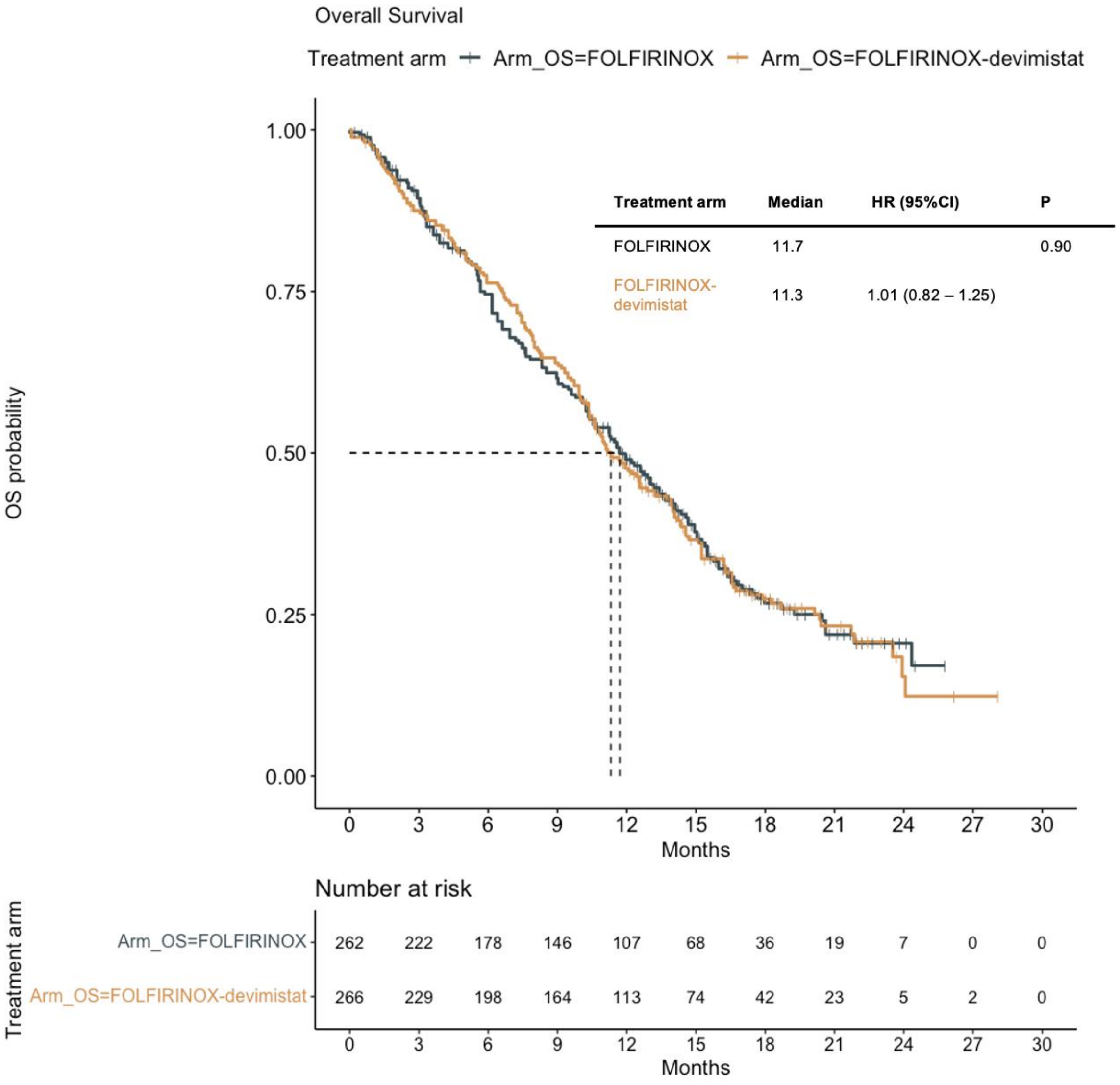

© 2023 Nichetti F et al. *JAMA Network Open.*

RESOLVE<br><br>PFS	The original figure may be found in the primary trial manuscript. Tempero M, Oh DY, Tabernero J et Al. Ibrutinib in combination with nab-paclitaxel and gemcitabine for first-line treatment of patients with metastatic pancreatic adenocarcinoma: phase III RESOLVE study. Ann Oncol. 2021 May;32(5):600-608. doi: 10.1016/j.annonc.2021.01.070. Epub 2021 Feb 1. PMID: 33539945.<br><br>Figure 2B	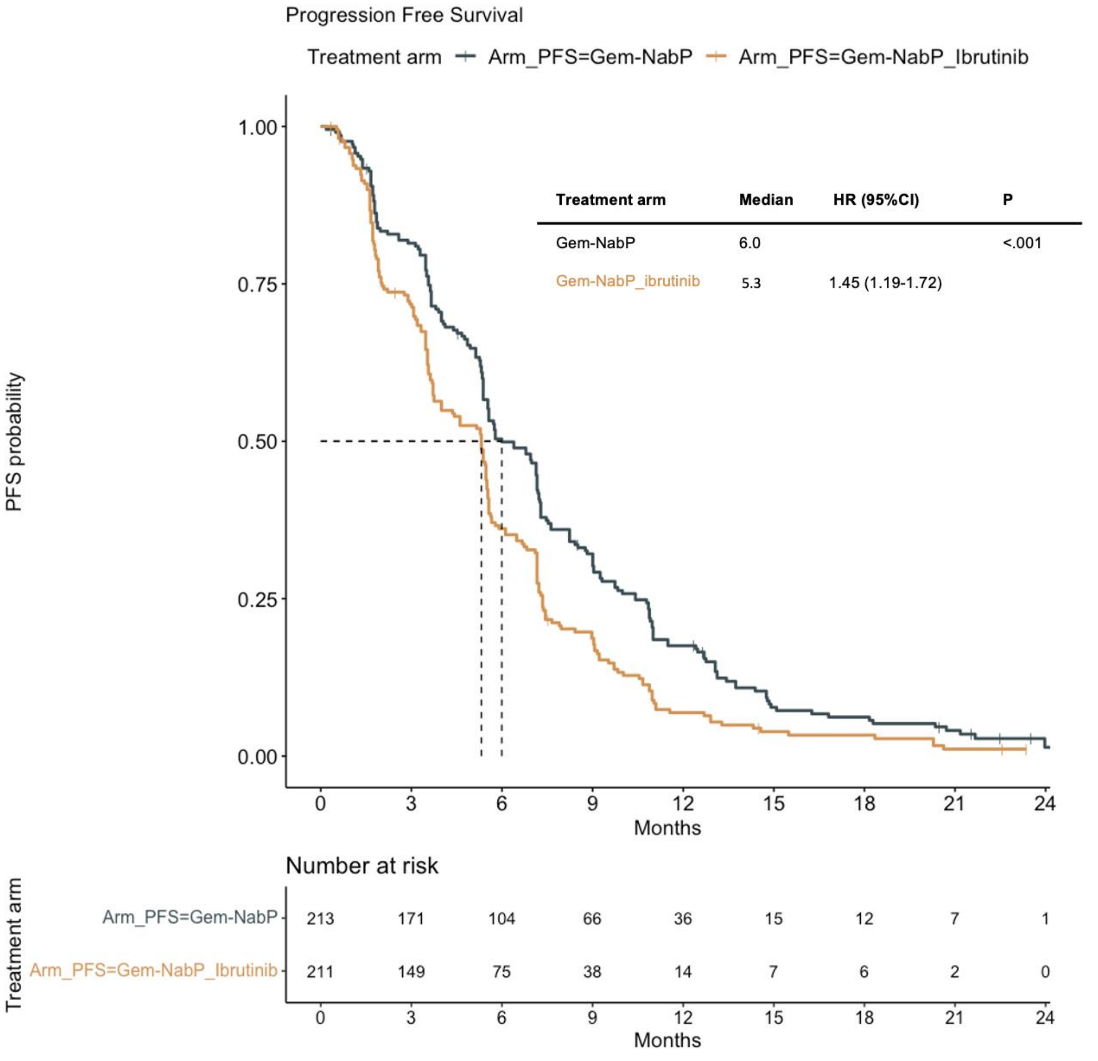

© 2023 Nichetti F et al. *JAMA Network Open.*

RESOLVE<br><br>OS	The original figure may be found in the primary trial manuscript. Tempero M, Oh DY, Tabernero J et Al. Ibrutinib in combination with nab-paclitaxel and gemcitabine for first-line treatment of patients with metastatic pancreatic adenocarcinoma: phase III RESOLVE study. Ann Oncol. 2021 May;32(5):600-608. doi: 10.1016/j.annonc.2021.01.070. Epub 2021 Feb 1. PMID: 33539945.<br><br>Figure 2A	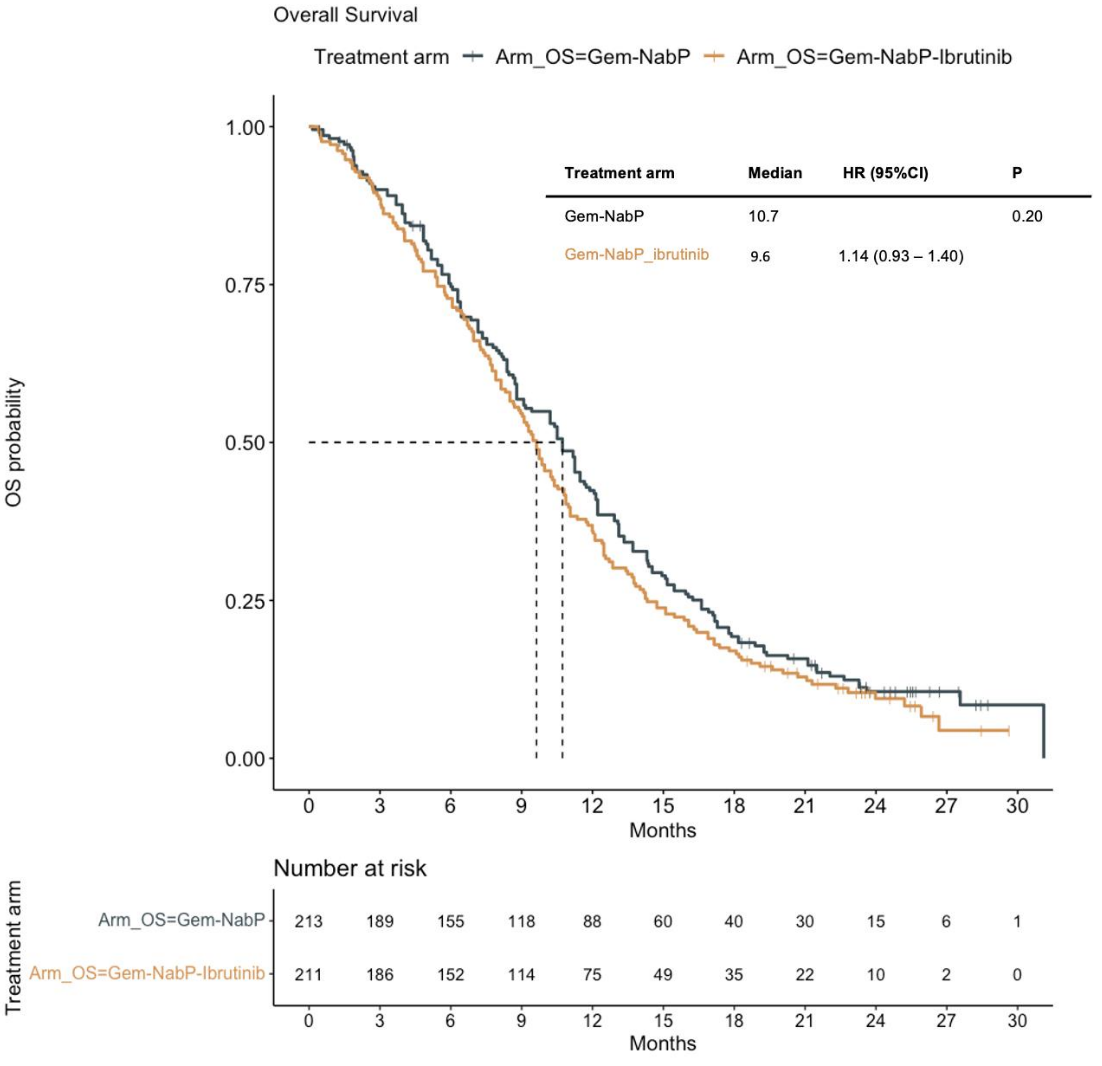

© 2023 Nichetti F et al. *JAMA Network Open.*

CanStem111P<br><br>PFS	The original figure may be found in the primary trial manuscript.<br>Bekaii-Saab T, Okusaka T, Goldstein D, et Al. Napabucasin plus nab-paclitaxel with gemcitabine versus nab-paclitaxel with gemcitabine in previously untreated metastatic pancreatic adenocarcinoma: an adaptive multicentre, randomised, open-label, phase 3, superiority trial. EClinicalMedicine. 2023 Mar 16;58:101897. doi: 10.1016/j.eclinm.2023.101897. PMID: 36969338; PMCID: PMC10036520.<br><br>Figure 2B	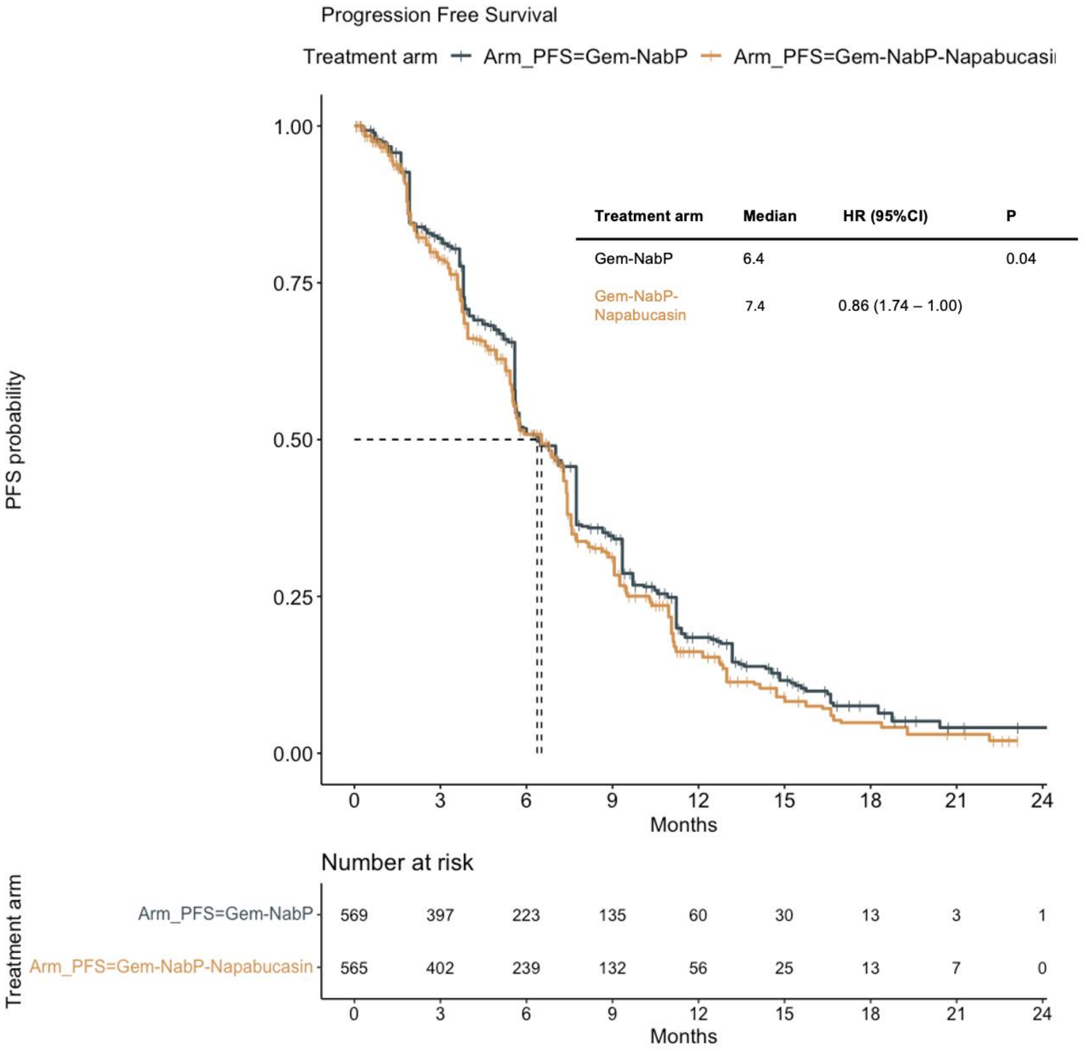

© 2023 Nichetti F et al. *JAMA Network Open.*

CanStem111P<br><br>OS	The original figure may be found in the primary trial manuscript.<br>Bekaii-Saab T, Okusaka T, Goldstein D, et Al. Napabucasin plus nab-paclitaxel with gemcitabine versus nab-paclitaxel with gemcitabine in previously untreated metastatic pancreatic adenocarcinoma: an adaptive multicentre, randomised, open-label, phase 3, superiority trial. EClinicalMedicine. 2023 Mar 16;58:101897. doi: 10.1016/j.eclinm.2023.101897. PMID: 36969338; PMCID: PMC10036520.<br><br>Figure 2A	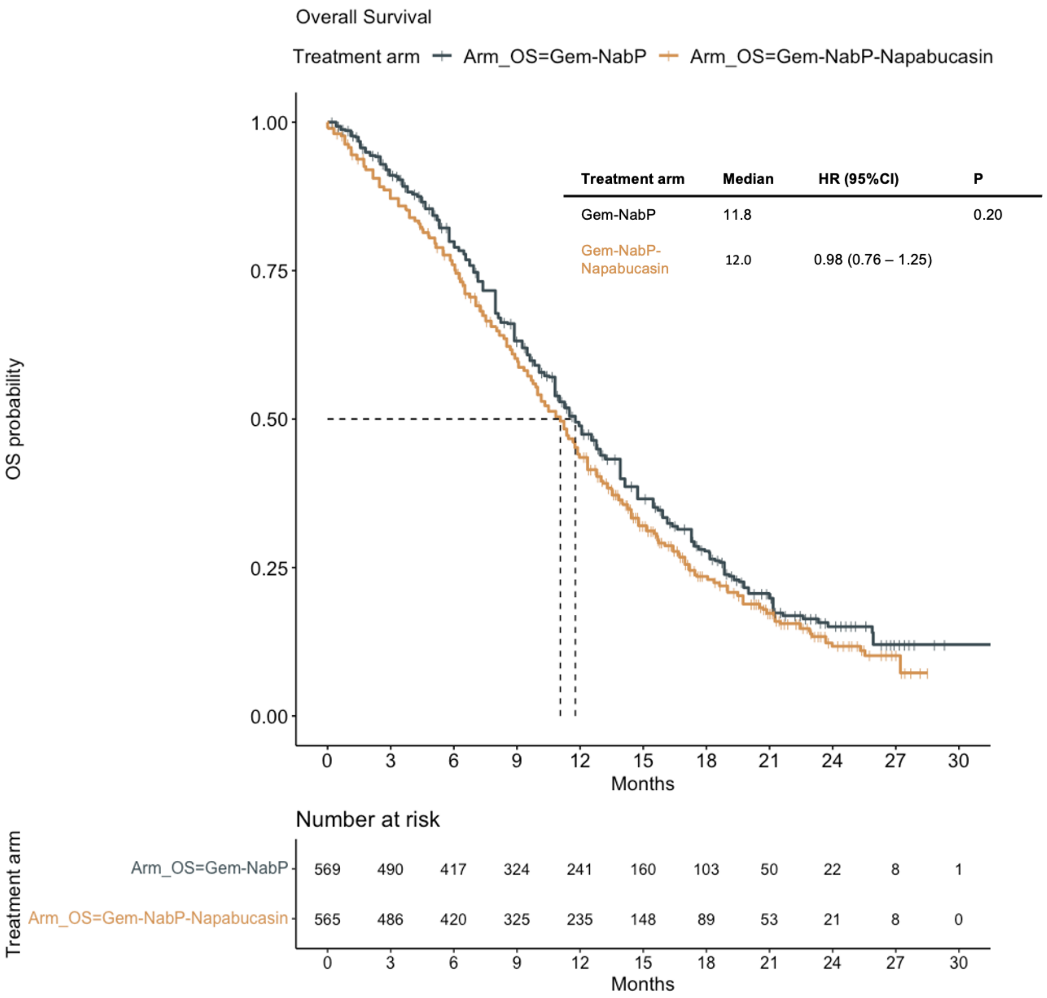

Abbreviations: CI: confidence interval; FOLFIRINOX: irinotecan + oxaliplatin + folinic acid + 5-fluoruracil; Gem-NabP: gemcitabine + NabPaclitaxel; HR: hazard ratio; NALIRIFOX: liposomal irinotecan + oxaliplatin + folinic acid + 5-fluoruracil.

© 2023 Nichetti F et al. *JAMA Network Open.*

Supplementary Table 4: Survival analysis and adjusted Cox regression models for overall survival and Progression Free Survival.

**Overall Survival**				
**Treatment arm**	**n**	**Median (95%CI)**	**HR (95%CI)**	**p**
NALIRIFOX	383	7.4 (6.1 - 7.7)	ref	
FOLFIRINOX	433	7.3 (6.5 - 7.9)	1.21 (0.86 - 1.70)	0.28
Gem-NabP	1765	5.7 (5.6 - 6.1)	1.45 (1.22 - 1.73)	<.001
			vs FOLFIRINOX 1.20 (0.88 - 1.64)	0.24
**Progression Free Survival**				
NALIRIFOX	383	11.1 (10.1 - 12.3)	ref	
FOLFIRINOX	433	11.7 (10.4 - 13.0)	1.06 (0.81 - 1.39)	0.65
Gem-NabP	1765	10.4 (9.8 - 10.8)	1.18 (1.00 - 1.39)	0.05
			vs FOLFIRINOX 1.11 (0.88 - 1.39)	0.37

Abbreviations: CI: confidence interval; FOLFIRINOX: 5-fluorouracil, leucovorin, irinotecan and oxaliplatin; Gem-NabP: gemcitabine plus Nab-Paclitaxel; HR: hazard ratio; NALIRIFOX: 5-fluorouracil, leucovorin, liposomal irinotecan and oxaliplatin;

© 2023 Nichetti F et al. *JAMA Network Open.*

Supplementary Table 5: 6- and 12-months OS and PFS.

Treatment Arm	6-months OS % (95% CI)	12-months OS % (95% CI)	p*	6-months PFS % (95% CI)	12-months PFS % (95% CI)	p*
**NALIRIFOX**	73 (68 - 77)	46 (41 - 51)	0.30	56 (51 - 62)	27 (22 - 33)	0.02
**FOLFIRINOX**	75 (71 - 80)	49 (44 - 54)		58 (53 - 63)	23 (18 - 29)	
**Gem-NabP**	73 (71 - 75)	43 (40 - 45)		48 (45 - 50)	17 (15 - 19)	

*Peto & Peto modification of Gehan-Wilcoxon test.

Abbreviations: CI: confidence interval; FOLFIRINOX: 5-fluorouracil, leucovorin, irinotecan and oxaliplatin; Gem-NabP: gemcitabine plus Nab-Paclitaxel; NALIRIFOX: 5-fluorouracil, leucovorin, liposomal irinotecan and oxaliplatin; OS: overall survival; PFS: progression-free survival.

© 2023 Nichetti F et al. *JAMA Network Open.*

Supplementary Table 6: Power analysis.

Outcome	Experimental Arm	Control Arm	Experimental Arm n° of patients	Control Arm n° of patients	HR	Power of upstream analyses	Total n° of patients (in 1:1 random) required to attain a power of 0.8
**OS**	NALIRIFOX	FOLFIRINOX or Gem-NabP	383	2198	0.85	0.65	1714
**OS**	NALIRIFOX	Gem-NabP	383	1765	0.85	0.64	1690
**PFS**	NALIRIFOX	FOLFIRINOX or Gem-NabP	383	2198	0.69	0.99	374
**PFS**	NALIRIFOX	Gem-NabP	383	1756	0.70	0.99	394
**Outcome**	**Experimental Arm**	**Control Arm**	**Experimental Arm n° of patients**	**Control Arm n° of patients**	**HR**	**Non-inferiority HR evaluable with 80% power**	**Total n° of patients (in 1:1 random) required to test non-inferiority**
**OS**	NALIRIFOX	FOLFIRINOX	383	433	1.02	1.23	1433
**PFS**	NALIRIFOX	FOLFIRINOX	383	433	0.95	1.23	287

Abbreviations: FOLFIRINOX: 5-fluorouracil, leucovorin, irinotecan and oxaliplatin; Gem-NabP: gemcitabine plus Nab-Paclitaxel; HR: hazard ratio; NALIRIFOX: 5-fluorouracil, leucovorin, liposomal irinotecan and oxaliplatin; OS: overall survival; PFS: progression-free survival.

© 2023 Nichetti F et al. *JAMA Network Open.*

Supplementary Table 7. Results of logistic regression analysis for G≥3 toxicities according to pooled treatment regimens.

Toxicity	regimen	Rates (%)	Odds Ratio	conf.low	conf.high	p-value
**Anemia**	NALIRIFOX *vs* FOLFIRINOX	10.5 vs 11.2	0.99	0.56	1.75	0.97
	NALIRIFOX vs Gem-NabP	10.5 vs 18.8	0.55	0.37	0.82	0.003
	Gem-NabP vs FOLFIRINOX	18.0 vs 11.2	1.81	1.15	2.84	0.01
**Neutrophil count decreased**†	NALIRIFOX *vs* FOLFIRINOX	23.8 vs 30.8	0.66	0.28	1.55	0.34
	NALIRIFOX *vs* Gem-NabP	23.8 vs 34.6	0.51	0.37	0.70	<.001
	Gem-NabP vs FOLFIRINOX	34.6 vs 30.8	1.30	0.58	2.91	0.52
**Febrile Neutropenia**	NALIRIFOX *vs* FOLFIRINOX	2.4 vs 7.7	0.30	0.14	0.63	0.002
	NALIRIFOX *vs* Gem-NabP	2.4 vs 3.0	0.80	0.39	1.63	0.53
	Gem-NabP vs FOLFIRINOX	3.0 vs 7.7	0.37	0.24	0.59	<.001
**Platelet count decreased***	NALIRIFOX *vs* FOLFIRINOX	1.6 vs 11.8	5.13	1.78	14.77	0.002
	NALIRIFOX *vs* Gem-NabP	1.6 vs 10.8	4.92	2.00	12.13	0.001
	Gem-NabP vs FOLFIRINOX	10.8 vs 11.8	1.04	0.57	1.91	0.89
**Diarrhea***	NALIRIFOX *vs* FOLFIRINOX	20.3 vs 16.8	1.36	0.72	2.58	0.35
	NALIRIFOX *vs* Gem-NabP	20.3 vs 5.7	4.39	2.77	6.98	<.001
	Gem-NabP vs FOLFIRINOX	5.7 vs 16.8	0.32	0.20	0.51	<.001
**Peripheal neuropathy***†	NALIRIFOX *vs* FOLFIRINOX	6.8 vs 9.0	0.90	0.35	2.32	0.83
	NALIRIFOX *vs* Gem-NabP	6.8 vs 12.1	0.70	0.41	1.18	0.18
	Gem-NabP vs FOLFIRINOX	12.1 vs 9.0	1.29	0.55	3.04	0.56
**Vomit***	NALIRIFOX vs FOLFIRINOX	7.0 vs 14.5	0.45	0.25	0.81	0.007
	NALIRIFOX *vs* Gem-NabP	7.0 vs 2.4	3.12	1.60	6.05	0.001

© 2023 Nichetti F et al. *JAMA Network Open.*

	Gem-NabP vs FOLFIRINOX	2.4 vs 14.5	0.14	0.07	0.28	<.001
**Fatigue*†**	NALIRIFOX *vs* FOLFIRINOX	15.1 vs 16.5	0.90	0.35	2.32	0.83
	NALIRIFOX *vs* Gem-NabP	15.1 vs 14.5	0.70	0.41	1.18	0.18
	Gem-NabP vs FOLFIRINOX	14.5 vs 16.5	1.29	0.55	3.04	0.56

* The following toxicities were not detailed in all trials. In detail:
- Platelet count decreased and Fatigue rates were not available in CanStem111P trial results;
- Diarrhea rates were not available in HALO trial results;
- Peripheral neuropathy rates were not available in CanStem111P, HALO and AVENGER500 trial results;
- Vomit rates were not available in CanStem111P, MPACT, HALO and AVENGER500 trial results.

† Equivalent terms reported separately in original reports were pooled before the analysis, including “Neutrophil count decreased” and “Neutropenia”, “Peripheral neuropathy” and “Peripheral sensory neuropathy”, and “Fatigue” and “Asthenia”.

Abbreviations: conf.low/high= lower/upper bound of 95% confidence interval; FOLFIRINOX: irinotecan + oxaliplatin + folinic acid + 5-fluoruracil; Gem-NabP: gemcitabine + NabPaclitaxel; NALIRIFOX: liposomal irinotecan + oxaliplatin + folinic acid + 5-fluoruracil.

© 2023 Nichetti F et al. *JAMA Network Open.*

Supplementary Figures

Supplementary Figure 1. PRISMA Flow Diagram

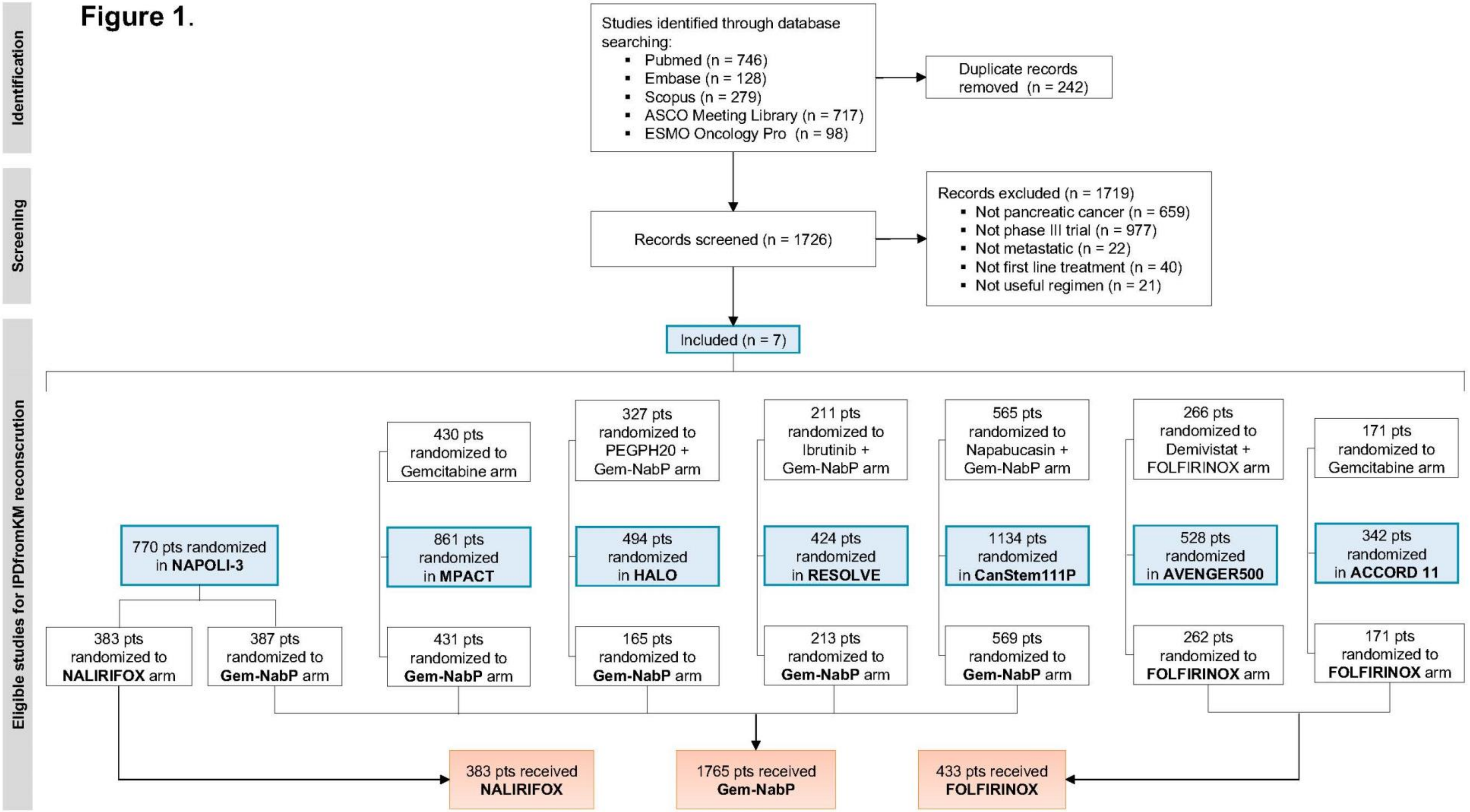

© 2023 Nichetti F et al. *JAMA Network Open.*

Supplementary Figure 2A-B. Reconstructed Kaplan-Meier plots for (A) Progression-Free Survival and (B) Overall Survival according to first line regimen with follow up censored at the time of the shortest follow up among included studies.

Supplementary Figure 1 Part A.

S1A

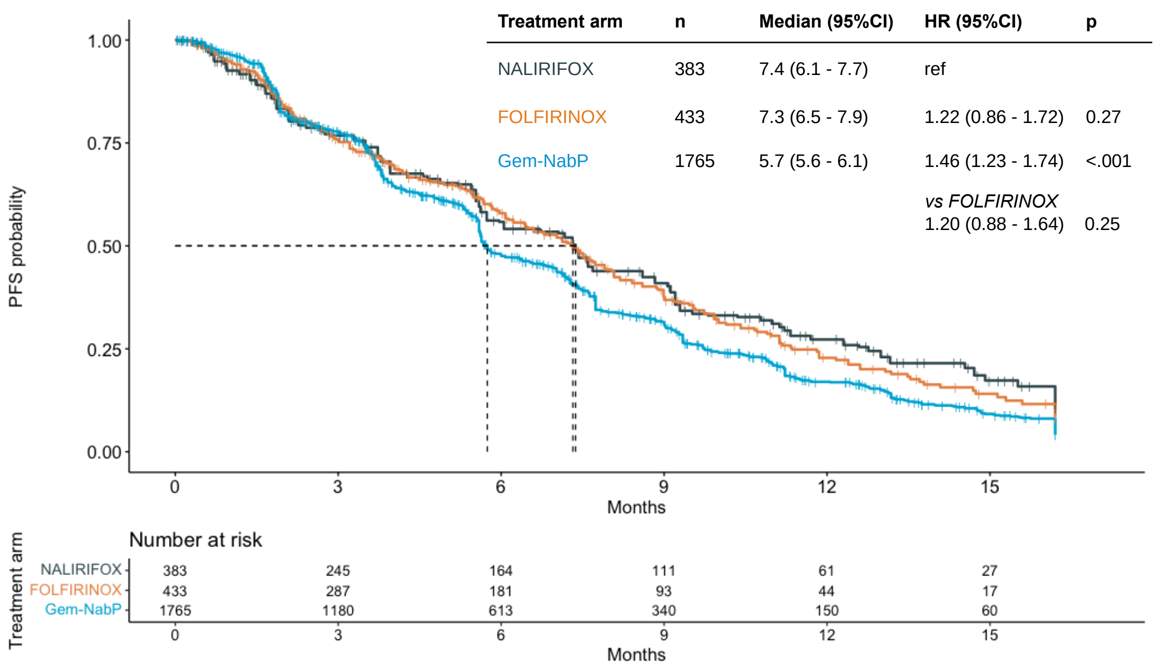

© 2023 Nichetti F et al. *JAMA Network Open.*

Supplementary Figure 1 Part B.

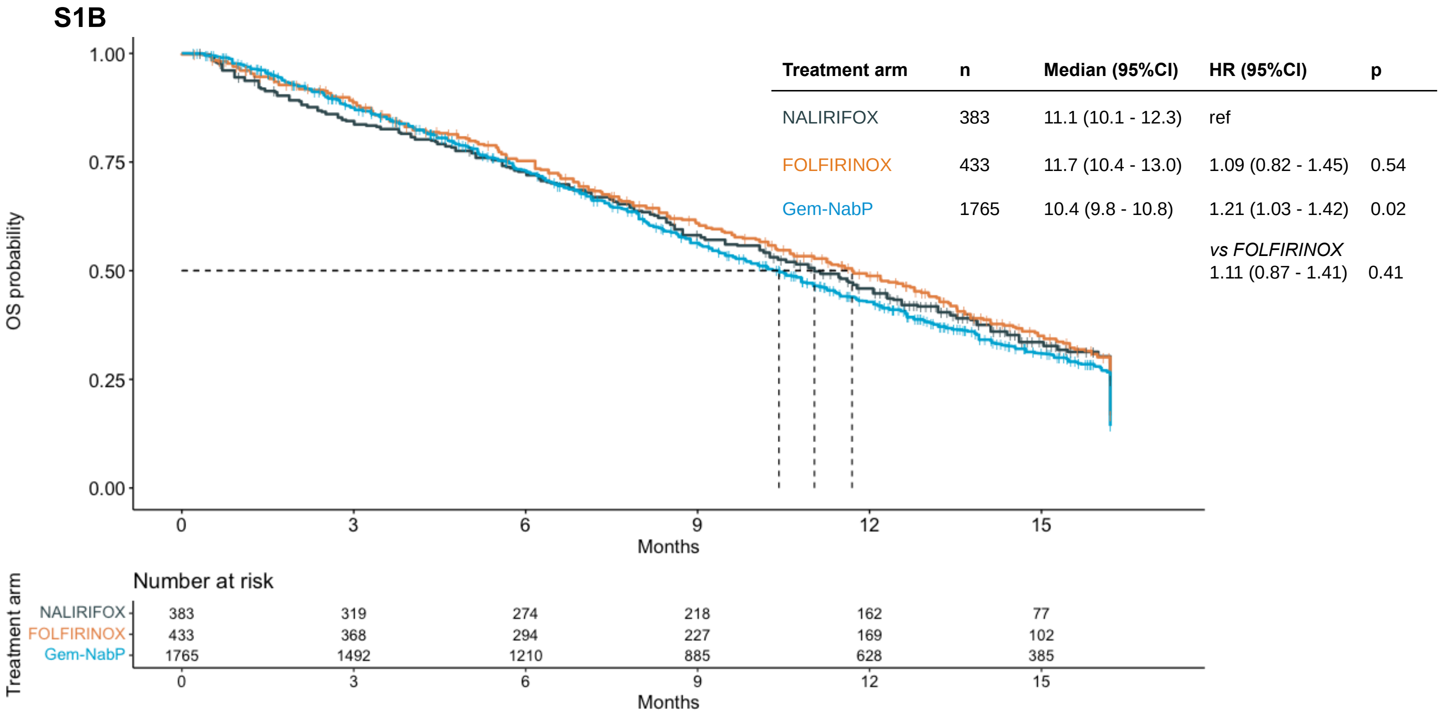

Abbreviations: CI: confidence interval; FOLFIRINOX: irinotecan + oxaliplatin + folinic acid + 5-fluoruracil; Gem-NabP: gemcitabine + NabPaclitaxel; HR: hazard ratio; NALIRIFOX: liposomal irinotecan + oxaliplatin + folinic acid + 5-fluoruracil.

© 2023 Nichetti F et al. *JAMA Network Open.*

Supplementary Figure 3A-B. Forest plots of Progression-Free Survival (S1B) and Overall Survival (S1B) in the network meta-analysis. NALIRIFOX is adopted as reference.

Supplementary Figure 2 Parts A-B.

S2A

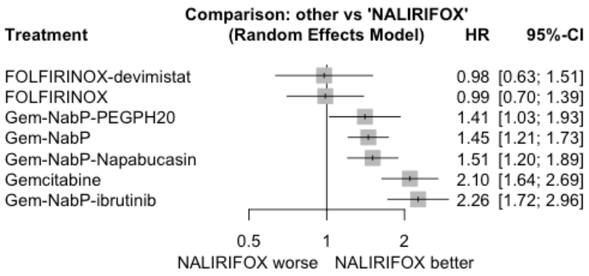

S2B

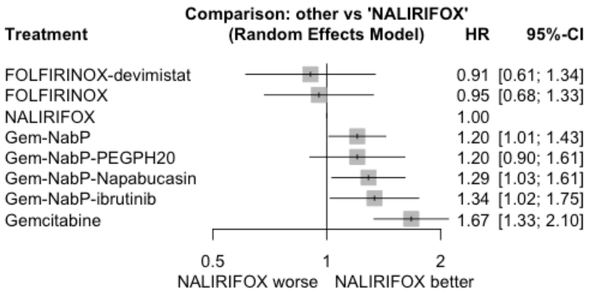

Abbreviations: CI: confidence interval; FOLFIRINOX: irinotecan + oxaliplatin + folinic acid + 5-fluoruracil; Gem-NabP: gemcitabine + NabPaclitaxel; HR: hazard ratio; NALIRIFOX: liposomal irinotecan + oxaliplatin + folinic acid + 5-fluoruracil.

© 2023 Nichetti F et al. *JAMA Network Open.*

Supplementary Figure 4A-B. Forest plots of Progression-Free Survival (S1A) and Overall Survival (S1B) in patients treated with gemcitabine plus NabPaclitaxel according to the respective clinical trial.

Supplementary Figure 3 Parts A-B.

S3A

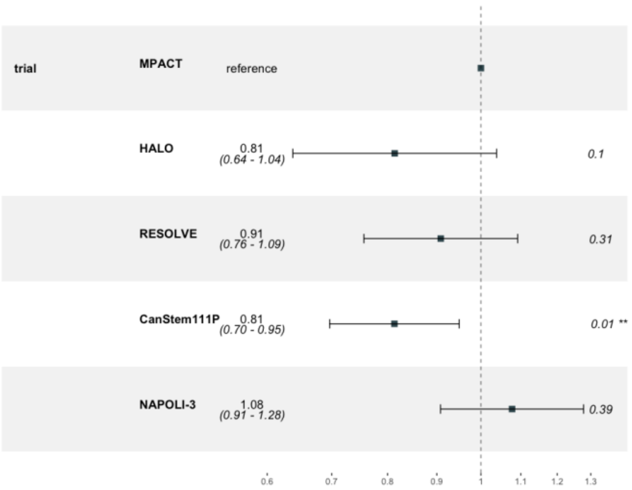

S3B

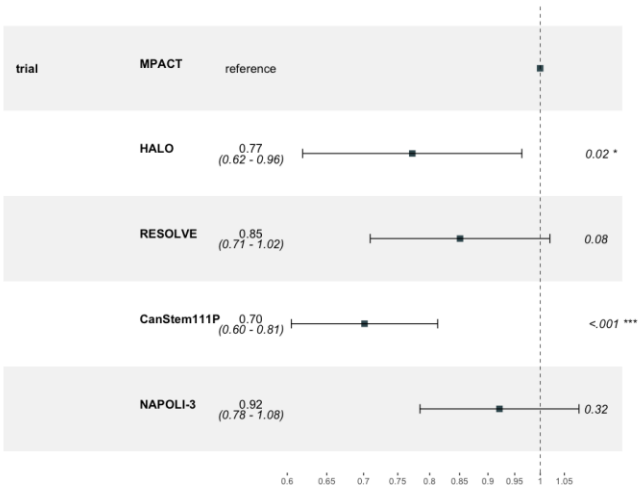

© 2023 Nichetti F et al. *JAMA Network Open.*

Supplementary Figure 5A-B. Barplots reporting best response (A) and overall response rate (B) according to the pooled treatment arms. Chi-squared tests pvalues are reported in (A), while OR (adjusted with clinical trials as random effect variable), together with corresponding 95%CI and p values are reported in (B).

Supplementary Figure 4 Part A-B.

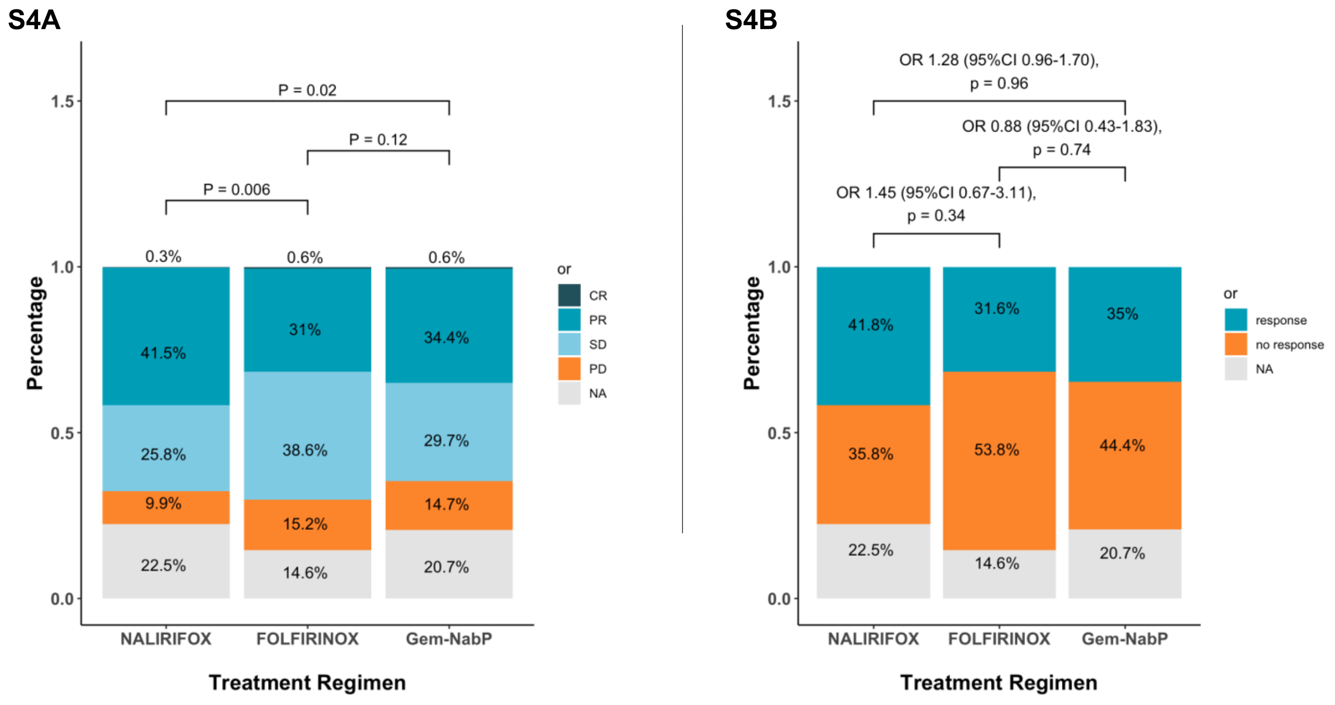

Abbreviations: CI: confidence interval; CR: complete response; FOLFIRINOX: irinotecan + oxaliplatin + folinic acid + 5-fluoruracil; Gem-NabP: gemcitabine + NabPaclitaxel; HR: hazard ratio; NA: not available; NALIRIFOX: liposomal irinotecan + oxaliplatin + folinic acid + 5-fluoruracil; OR: odds ratio; PD: progressive disease; PR: partial response; SD: stable disease.

© 2023 Nichetti F et al. *JAMA Network Open.*
