# Supplementary material for: NALIRIFOX, FOLFIRINOX, and Gemcitabine With Nab-Paclitaxel as First-Line Chemotherapy for Metastatic Pancreatic Cancer: A Systematic Review and Meta-Analysis
Source: JAMA Netw Open. 2024 Jan 8;7(1):e2350756. doi: 10.1001/jamanetworkopen.2023.50756 (PMC10774994; doi:10.1001/jamanetworkopen.2023.50756)
Supplement: Supplement 2. — Data Sharing Statement [file jamanetwopen-e2350756-s002.pdf]

Data Sharing Statement

Nichetti. NALIRIFOX, FOLFIRINOX, and Gemcitabine With Nab-Paclitaxel as First-Line Chemotherapy for Metastatic Pancreatic Cancer. *JAMA Netw Open*. Published January 08, 2024. doi:10.1001/jamanetworkopen.2023.50756

Data
Data available: No

Additional Information
Explanation for why data not available: Data are already publicly available
